# Deciphering plant stress resilience in different millet crops: from multi-omics discoveries to predictive breeding for climate-resilient crop enhancement

**DOI:** 10.3389/fpls.2026.1971866

**Published:** 2026-10-01

**Authors:** Mahalakshmi Mani, Poonguzhali Subramanian, Sourav Paramanik, Madhusmita Dishri

**Affiliations:** VIT School of Agricultural Innovations and Advanced Learning (VAIAL), Vellore Institute of Technology, Vellore, Tamil Nadu, India

**Keywords:** millets, multi-omics, abiotic stress, predictive breeding, high-throughput phenotyping

## Abstract

Global climatic and abiotic stresses severely affect agricultural production, while millets are stress-tolerant and nutritious cereal crops adapted to diverse environments. Although single-omics technologies, including genomics, transcriptomics, proteomics, metabolomics, epigenomics, ionomics, and phenomics, have substantially advanced our understanding of individual components of plant stress responses, each approach captures only one molecular layer and therefore cannot fully resolve the interconnected regulatory networks that govern complex stress adaption. The challenge now is to bring together these widely varied molecular data to make them biologically meaningful and to be able to predict genotype-phenotype relationships under varying field conditions. This review critically covers recent developments in multi-omics technologies that have been used to uncover abiotic stress tolerance mechanisms, and how they can be complementary to identify stress-responsive genes, regulatory pathways, stress markers, and adaptive metabolic processes. Additionally, the review covers recent developments in AI and ML integration with multi-omics data, predictive modeling, and precision breeding to enhance the identification of better genotypes and breeding decisions. Finally, future prospects for the integration of multi-omics, AI analytics, digital phenotyping and breeding pipelines for next-generation cultivars of millets for future climate change are outlined, which will enable the improvement of current cultivars with higher resilience, yield and nutritional value, while simultaneously supporting sustainable agriculture.

## 1 Introduction

Climate change poses unprecedented challenges to global agriculture, causing environmental stresses to crop production to become more frequent, severe and unpredictable. The frequency of rising temperatures, irregular precipitation, long dry periods, salinity intrusion, heat waves, flooding and nutrient deficits is mounting and occurring as a package. These multi-and synergistic effects of stress limit plant growth, productivity and grain quality, jeopardizing food and nutritional security globally (Ortiz-Bobea et al., 2021). While land availability is declining and environmental conditions become more challenging, agricultural productivity needs to grow significantly by 2050 to support a global population of almost 10 billion. Climate resistance of crops by developing climate-resilient varieties that can sustain stable yields under multiple environmental stresses has thus emerged as one of the greatest priorities in crop improvement (Sinha et al., 2021; Choudhary et al., 2023). Millets, being highly resilient to stress in agroecological conditions, have been identified as key crops in climate-smart agriculture among the cereal crops. The species are also highly drought-resistant, heat-tolerant, salt-tolerant, and adapted to nutrient-poor soils and have relatively low production requirements compared to major cereals (Crossa et al., 2017; Saxena et al., 2018).

Natural genetic variation has been harnessed for stress tolerance in the process of crop improvement through conventional breeding, which has greatly helped improve crops. Most abiotic stress tolerance traits are polygenic, with many genes contributing small individual effects and strong developmental stage, environment and genotype × environment interactions (Varshney et al., 2021). With the increased incidence of multiple abiotic stresses, the breeding process is further complicated, as molecular changes that enable it to withstand one stress alone may not be beneficial or may even have an inhibitory effect when faced with multiple stress situations (Zandalinas et al., 2021). Hence, traditional selection methods may not be adequate to address the complex regulation of climate resilience (Varshney et al., 2021). High-throughput omics technologies have revolutionized plant biology, providing detailed characterization of biological processes at several molecular levels (Yang et al., 2021; Raza et al., 2024). Advances in genomics, transcriptomics, proteomics, metabolomics, phenomics, epigenomics, ionomics, and other emerging platforms have expedited gene discovery, regulatory element discovery, protein discovery, metabolite discovery, and physiological traits associated with stress adaptation (Yang et al., 2021; Raza et al., 2024).

To overcome these limitations, there is a shift in paradigm from reductionist analyses to more comprehensive systems biology approaches by combining information from multiple omics layers to build comprehensive models of plant stress responses (Hasin et al., 2017; Huang et al., 2023). Combining these various levels of information allows for a multi-omics integration that enables simultaneous interrogation of genomic variation, transcriptional regulation, protein abundance, metabolite dynamics, epigenetic modifications, elemental homeostasis, and high-throughput phenotypes, thereby facilitating a holistic understanding of stress adaptation (Hasin et al., 2017; Yang et al., 2021). These integrated approaches enhance the identification of key regulatory hubs, molecular biomarkers, and candidate genes that can be leveraged in marker-assisted breeding, genomic selection, genome editing, and precision breeding programs, offering improved opportunities to utilize the molecular mechanisms of abiotic stress-response pathways in early selection for improved stress-resilience traits in crop cultivars (Varshney et al., 2021; Raza et al., 2024). More importantly, systems-level analyses open the door to explore the complex interactions that result from combined abiotic stresses, which are still not known to the extent of their agricultural relevance (Zandalinas et al., 2021). The use of multi-omics research has continued to broaden with the rapid advancement of artificial intelligence (AI), machine learning (ML), deep learning (DL), and advanced computational biology (Jamil et al., 2020).

These new technologies offer unparalleled possibilities to develop millet varieties that are more resilient to climate fluctuations and can ensure yields without any compromises (Raza et al., 2024). Although significant advances have been made, there are several key obstacles that need to be addressed for the practical application of multi-omics-assisted breeding. Despite these advances, challenges such as limited interoperability between omics datasets, poor validation of molecular discoveries under field conditions, a lack of studies investigating combined abiotic stresses, shortage of standardized computational pipelines and the absence of a predictive framework for linking molecular variation to agronomic performance are significant bottlenecks (Jamil et al., 2020). In addition, the use of new technologies, such as single-cell omics, spatial transcriptomics, pan-genomics, graph genomes, digital twins, and explainable AI, in crop improvement remains in its infancy and is an important cutting-edge area for the future (Raza et al., 2024). This review not only summarizes single omics technologies, but also delivers a systems-level integrative, forward-looking outlook of plant stress biology by highlighting AI-enabled predictive breeding and integration of multi-omics technologies for plant improvement of climate resilience. The review critically analyzes the merits and the drawbacks of individual omics platforms, identifies new technologies and computational advances, outlines existing gaps in knowledge, and recommends future directions and applications of multi-omics, AI, and precision breeding to create resilient crop varieties for future sustainable agriculture (**Figure 1**).

**Figure 1:**
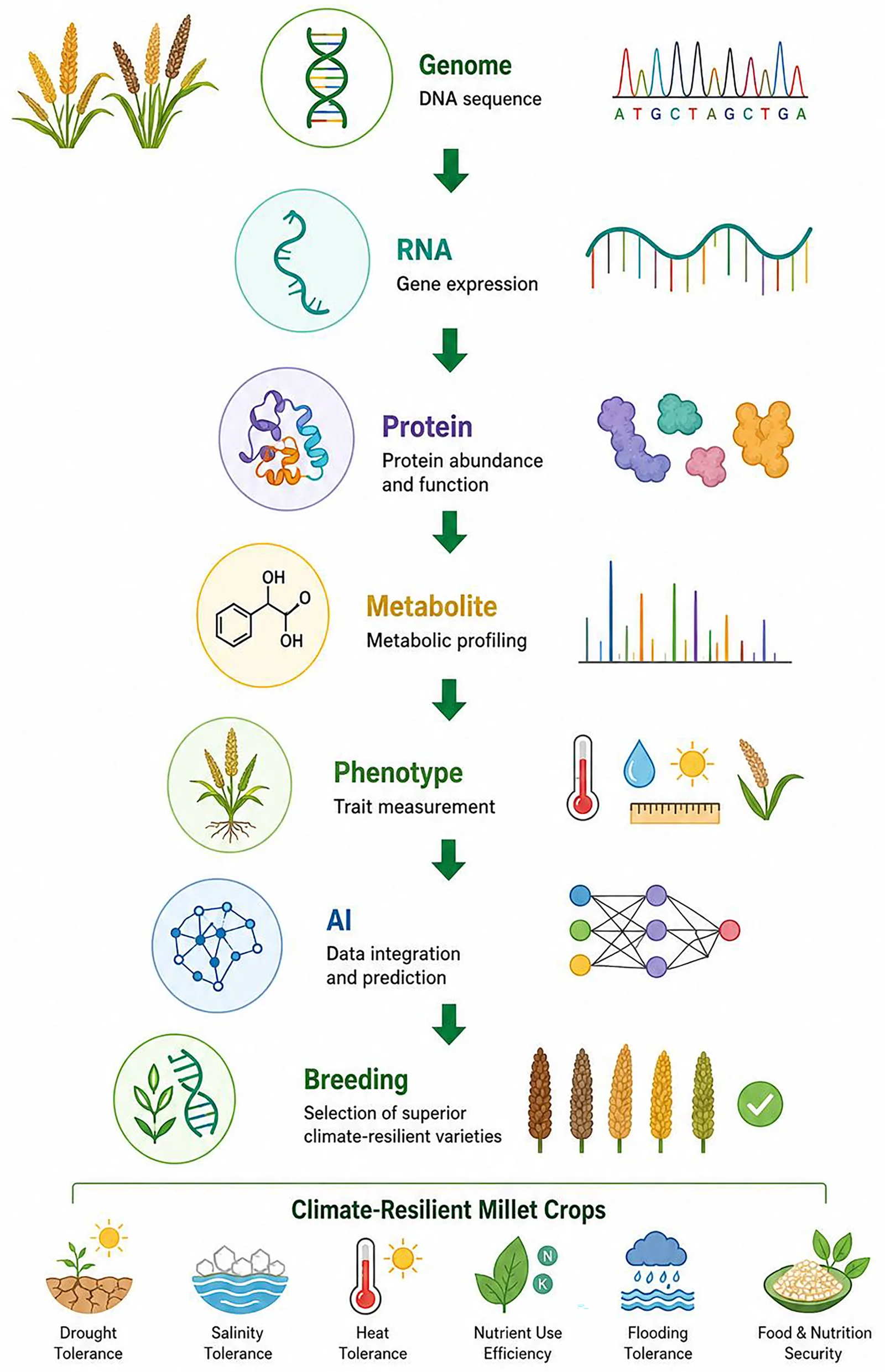
Multi-omics and AI-driven breeding framework for developing climate-resilient millet crops. The framework illustrates the integration of genomic, transcriptomic, proteomic, metabolomic, phenomic, and AI approaches to characterize molecular responses, identify stress-associated traits, and predict superior genotypes.

## 2 Molecular discoveries to digital twin-assisted predictive breeding in millet crops

Millets and related climate-resilient cereal crops are well adapted to dry, saline soils, heat stress, and low-input marginal environments, including pearl millet (*Pennisetum glaucum*), finger millet (*Eleusine coracana*), foxtail millet (*Setaria italica*), sorghum (*Sorghum bicolor*), proso millet (*Panicum miliaceum*), barnyard millet (*Echinochloa frumentacea*), little millet (*Panicum sumatrense)*, kodo millet (*Paspalum scrobiculatum*), and browntop millet (*Urochloa ramosa*). Building on the stress resilience of millet crops, recent advances in high-throughput molecular technologies have progressively shifted millet improvement from conventional trait-based selection toward molecularly informed and predictive breeding. Over the past two decades, research has progressed from genome characterization and molecular marker development to functional omics, multi-omics integration, AI-assisted prediction, and digital twin-based modeling (Jamil et al., 2020; Raza et al., 2024). This progression has enabled the investigation of stress adaptation at increasingly higher levels of biological resolution, from genomic variation and gene expression to proteins, metabolites, ion homeostasis and whole-plant phenotypes. The integration of these datasets provides a framework for identifying regulatory networks, candidate genes and biomarkers and for translating molecular information into predictive breeding decisions.

### 2.1 Genome-centric discovery

The first generation of plant omics was mainly genome characterization and development of molecular markers. In this era, quantitative trait locus (QTL) mapping, expressed sequence tag (EST) sequencing, simple sequence repeat (SSR) markers, amplified fragment length polymorphisms (AFLPs) and, subsequently, single nucleotide polymorphism (SNP) markers were used to identify genomic regions associated with drought tolerance, stay-green phenotype, flowering time, nutrient-use efficiency, and grain quality in millet species (Varshney et al., 2020). With the sequencing of the draft and reference genome assemblies of a few model and economically important millets, particularly foxtail millet and sorghum, the genomic resources of these crops were greatly expanded and comparative genomics among cereals was made possible (Bennetzen et al., 2012).

### 2.2 Functional omics and molecular characterization

During the second wave of plant omics, the field of functional genomics made significant progress with the use of transcriptomics, proteomics, metabolomics, epigenomics, ionomics, and phenomics (Yang et al., 2021; Raza et al., 2024). By using high-throughput RNA sequencing (RNA-seq), the stress-responsive genes, transcription factors, and regulatory pathways under drought, salinity, heat, and nutrient stress were identified genome-wide (Yang et al., 2021). Proteomic analyses using LC-MS/MS and quantitative mass spectrometry identified proteins and post-translational modifications (PTMs) whose abundance and functional states are not necessarily predictable from the levels of their corresponding transcripts (Kosová et al., 2018). At the same time, metabolomics showed dynamic metabolic reprogramming of osmo-protectants, antioxidants, phytohormones, amino acids and secondary metabolites, which are involved in stress adaptation (Razzaq et al., 2019). In this regard, investigations of epigenomic systems have identified the roles of DNA methylation, histone modifications, and chromatin remodeling in regulating stress memory, and have advanced understanding of nutrient acquisition and ion homeostasis under adverse environmental conditions through ionomics (Kumar and Mohapatra, 2021).

### 2.3 Multi-omics era: systems biology for climate-resilient

Recent research has moved beyond independent omics analysis to the integration of multiple omics, which combine genomic, transcriptomic, proteomic, metabolomic, epigenomic, ionomic, and phenomic data (Hasin et al., 2017; Raza et al., 2024). This systems biology approach can be used to reconstruct complex regulatory networks that regulate stress perception, signal transduction, metabolic adaptation, and phenotypic plasticity (Jamil et al., 2020). The integration of multiple omics has significantly enhanced the identification of drought tolerance, heat stress, nutrient-use efficiency, and grain nutritional quality candidate genes, biomarkers, regulatory hubs, and molecular pathways in millet crops (Yang et al., 2021). The availability of emerging genomic resources such as pangenomes, graph genomes and haplotype-resolved assemblies, long-read sequencing, and structural variation analysis has further increased the identification of adaptive alleles that are not present in single reference genomes (Varshney et al., 2020; Raza et al., 2024).

### 2.4 AI-assisted biology: transforming omics data into biological intelligence

With the development of multi-dimensional omics datasets, there have been significant challenges in computational analysis of omics data that traditional statistical analysis methods are not well equipped to handle (Jamil et al., 2020). High-dimensional biological data has been analyzed by AI, ML, and DL algorithms and various genotype-to-phenotype relationships have been found, with the ability to predict stress adaptation increasingly becoming more accurate for millets (Raza et al., 2024). Genomic prediction, candidate gene prioritization, image-based phenotyping, stress diagnosis, and multi-omics data integration are some of the relevant applications of Deep Neural Networks (DNNs), random forests, support vector machines, gradient boosting algorithms, Graph Neural Networks (GNNs), and transformer-based architectures (Jamil et al., 2020; Crossa et al., 2022; Montesinos López et al., 2022). AI systems coupled with remote sensing, Internet of Things (IoT) devices, Unmanned Aerial Vehicles (UAVs), phenotyping, and environmental monitoring provide the data streams and analytical capabilities needed to model genotype × environment interactions under field conditions (Varshney et al., 2020) (**Figure 2**). Although individual components of this approach are increasingly being applied, their integration into continuously updating predictive systems for millet breeding remains an emerging research area.

**Figure 2:**
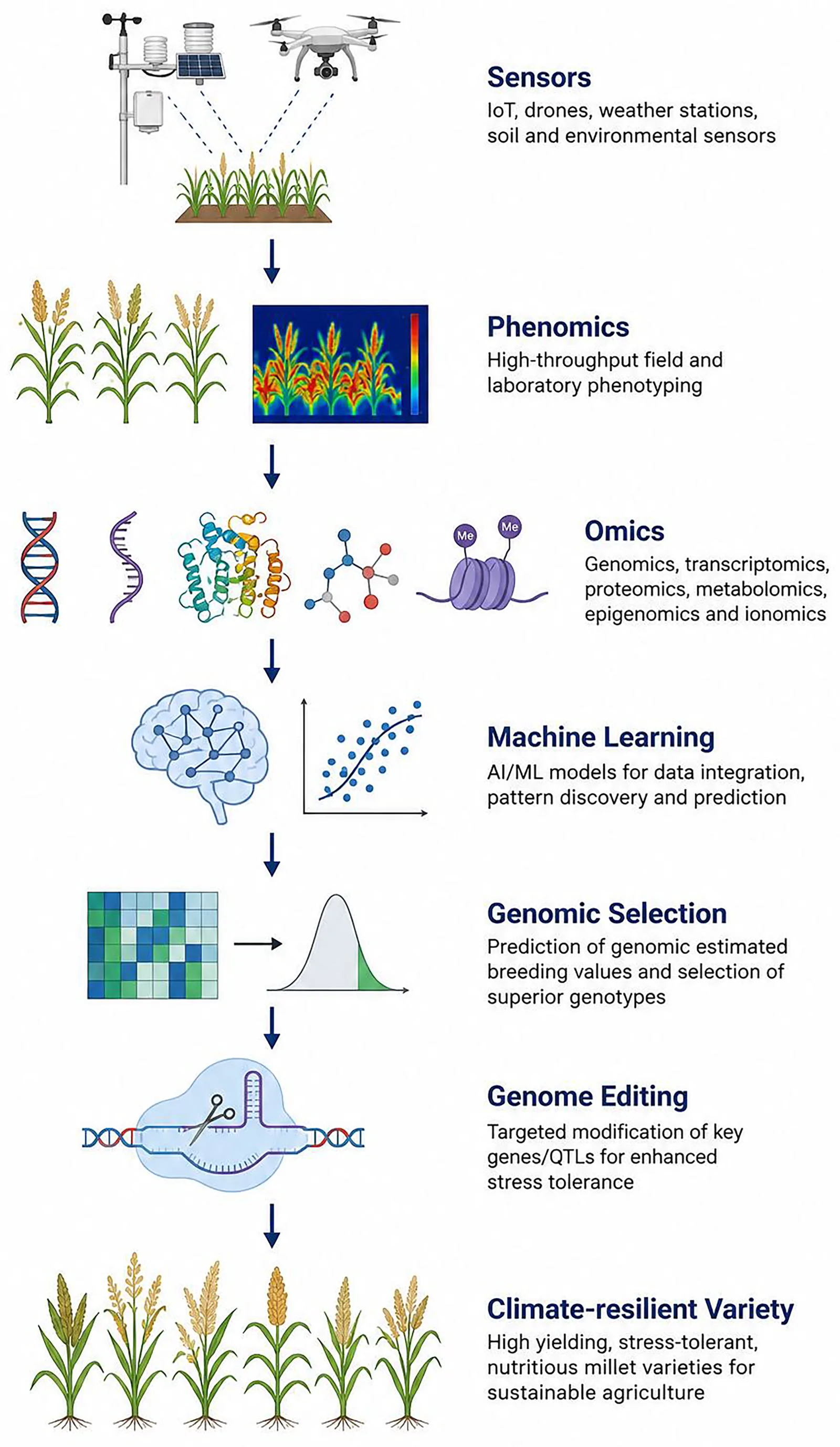
Integrated digital and molecular breeding framework for the development of climate-resilient millet varieties. The framework combines sensor technologies, IoT, drones, and environmental monitoring with high-throughput phenotyping to capture plant and field-level traits.

### 2.5 Predictive breeding: integrating multi-omics into crop improvement

The development of multi-omics technologies and their integration with genomic selection, genome-wide association studies (GWAS), speed breeding, genome editing, and AI-based prediction have led to the emergence of predictive breeding (Varshney et al., 2020; Raza et al., 2024). Predictive breeding is a breeding strategy that uses integrated molecular information to estimate breeding values, prioritize candidate genes, select superior parental combinations, and potentially accelerate breeding decisions by enabling earlier selection than would be possible when relying solely on extensive phenotypic evaluation (Jamil et al., 2020; Varshney et al., 2020). The capacity of genome editing technologies, including CRISPR/Cas systems, to introduce precise modifications in validated stress-responsive genes that were identified through multi-omics analysis further reinforces this framework (**Table 1**).

**Table 1:** Key abiotic stress-responsive genes identified in millet species and their functional roles in stress adaptation.

Gene	Species	Abiotic stress	Molecular function	Validated trait/phenotype	Experimental evidence	Reference
*DREB2A*	*Pennisetum glaucum*	Drought	AP2/ERF transcription factor regulating dehydration-responsive genes	Improved drought tolerance and osmotic adjustment	Gene expression analysis, transgenic validation	(Meena et al., 2022)
*NAC67*	*Eleusine coracana*	Drought	NAC transcription factor involved in stress-responsive signaling	Enhanced root development and water-use efficiency	Overexpression studies	(Rahman et al., 2016)
*EcNAC1*	*Eleusine coracana*	Drought, salinity	Transcriptional regulation of stress-inducible genes	Improved tolerance to water deficit	Functional characterization	(Kurowska and Daszkowska-Golec, 2023)
*PgP5CS*	*Pennisetum glaucum*	Drought, salinity	Proline biosynthesis (Δ¹-pyrroline-5-carboxylate synthetase)	Increased proline accumulation and osmotic protection	Expression profiling	(Sellamuthu et al., 2024)
*PgNHX1*	*Pennisetum glaucum*	Salinity	Vacuolar Na^+^/H^+^ antiporter maintaining ion homeostasis	Enhanced salt tolerance	Gene expression analysis	(Rajagopal et al., 2007)
*SOS1*	*Setaria italica*	Salinity	Plasma membrane Na^+^/H^+^ antiporter	Reduced sodium toxicity	Functional genomics	(Xie et al., 2022)
*HKT1;5*	*Setaria italica*	Salinity	Sodium transporter regulating Na^+^ exclusion	Improved ionic balance	QTL mapping and expression analysis	(Terfa et al., 2025)
*SiLEA14*	*Setaria italica*	Drought	Late embryogenesis abundant (LEA) protein protecting cellular structures	Increased dehydration tolerance	Transgenic studies	(Wang et al., 2014)
*SiCDPK24*	*Setaria italica*	Drought	Calcium-dependent protein kinase mediating ABA signaling	Enhanced drought response	Functional validation	(Yu et al., 2018)
*SiMYBS3*	*Setaria italica*	Drought	MYB transcription factor regulating ABA-responsive genes	Improved water deficit tolerance	Expression analysis	(Liu et al., 2023)
*SiWRKY genes*	*Setaria italica*	Drought	WRKY transcription factor involved in ROS scavenging	Enhanced oxidative stress tolerance	Gene expression studies	(Zhang et al., 2017)
*HSP70*	*Pennisetum glaucum*	Heat	Molecular chaperone preventing protein denaturation	Improved thermotolerance	Heat stress expression analysis	(Divya et al., 2019)
*HsfA2*	*Pennisetum glaucum*	Heat	Heat shock transcription factor regulating HSP genes	Increased heat stress tolerance	Functional genomics	(Singh et al., 2024)
*SOD*	*Eleusine coracana*	Drought	Superoxide dismutase scavenging reactive oxygen species	Improved antioxidant defense	Biochemical and molecular analysis	(Rakkammal et al., 2023)
*APX*	*Setaria italica*	Drought	Ascorbate peroxidase maintaining redox homeostasis	Reduced H_2_O_2_ accumulation	Expression profiling	(Sofo et al., 2015)

### 2.6 Digital twins: the next frontier in millet crop improvement

Digital twin technology represents a potential future framework for integrating heterogeneous biological and environmental information in crop improvement. In its broader engineering and agricultural context, a digital twin refers to a continuously updated virtual representation of a physical system that can integrate observations from multiple data sources and support simulation, monitoring, and scenario analysis (Jones et al., 2020; Liu et al., 2021). In crop systems, the concept could theoretically integrate genomic, transcriptomic, proteomic, metabolomic, phenomic, environmental, and management data to simulate crop growth and estimate genotype responses under contrasting environments. However, these capabilities should currently be regarded as a proposed framework for millet improvement rather than an established breeding technology. Several individual components required for a crop digital twin are already being developed or applied in millet research. These include genomic prediction, high-throughput and UAV-based phenotyping, remote sensing, environmental monitoring, and AI/ML-based analysis of genotype-to-phenotype relationships (Escribà-Gelonch et al., 2024). Such technologies can generate genotype, phenotype, and environmental datasets and can support prediction of selected traits or genotype × environment responses. Nevertheless, the integration of these components into a single continuously updating digital twin, capable of dynamically predicting the performance of millet genotypes and guiding breeding or management decisions in real time, has not yet been sufficiently validated in millet crops. Therefore, the digital-twin framework proposed in this review should be considered a forward-looking research model rather than a currently validated millet breeding platform. Its implementation can require standardized and interoperable multi-omics, phenotypic, environmental, and management datasets; robust genotype × environment × management models; continuous sensor-derived data streams, and independent multi-environment field validation. Once these components are experimentally validated and integrated, digital twins could potentially support scenario-based genotype evaluation, breeding decision support, and precision management in millet production (**Figure 3**).

**Figure 3:**
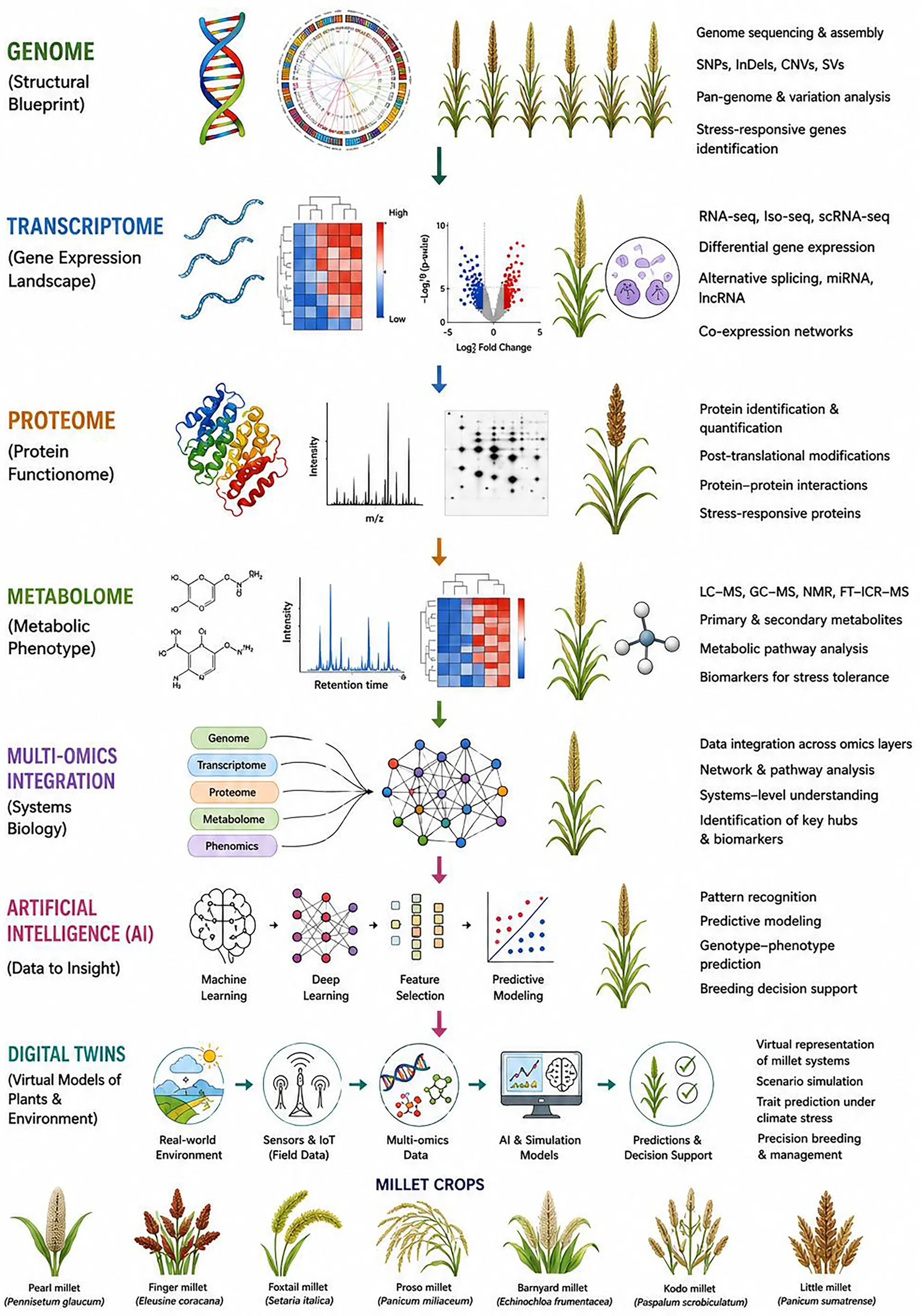
Multi-omics and AI-driven digital-twin framework for predictive breeding of climate-resilient millet crops. The framework illustrates a future integration of genomic, multi-omics, phenomic, environmental, sensor, and management data for genotype-to-phenotype modeling, scenario-based prediction, and breeding decision support.

### 2.7 Technology readiness level framework

To assess the translational maturity of omics technologies for millet breeding, we adapted the established nine-level TRL framework, from TRL 1 (basic principles) to TRL 9 (proven operational application), using a millet-breeding-specific interpretation. TRL assignments were based on five criteria: (1) technological maturity and reproducibility; (2) availability of millet-specific datasets and genomic resources; (3) experimental validation in millet germplasm; (4) validation under relevant or field conditions; and (5) integration into practical breeding activities, including trait discovery, marker development, genomic prediction, genotype evaluation, and selection. Ratings represent a qualitative synthesis of the literature reviewed rather than a numerical weighted score. In this framework, TRL 1–2 indicates basic principles or proposed applications; TRL 3–4, proof-of-concept and laboratory validation; TRL 5–6, validation in relevant biological or breeding environments; TRL 7, demonstration under realistic breeding or field-relevant conditions; TRL 8, mature and validated integration into breeding pipelines; and TRL 9, routine operational deployment with demonstrated utility. Thus, the TRLs in **Table 2** reflect the maturity of each technology’s application to climate-resilient millet breeding, rather than the intrinsic maturity of the omics platform. Higher ratings for genomics and phenomics reflect their established applications in millet improvement, whereas emerging approaches such as single-cell/spatial multi-omics, glycomics, secretomics, fluxomics, and redoxomics receive lower ratings due to limited millet-specific datasets, validation, standardized workflows, and breeding integration.

**Table 2:** Overview of omics technologies for climate-resilient millet breeding: advantages, limitations, applications, and millet breeding-specific technology readiness levels (TRLs).

Omics technology	Current TRL	Millet breeding specific basis	Reference
Genomics	8	Mature sequencing, genome assembly, GWAS, QTL mapping, and genomic-selection workflows; maturity is heterogeneous across millet species because some orphan millets still have incomplete reference resources and limited functional validation.	(Maharajan et al., 2024)
Transcriptomics	7-8	RNA-seq and stress-responsive gene discovery are well established in several millet species, but translation of transcriptomic discoveries into routine breeding remains limited.	(Sanku et al., 2024)
Proteomics	7	Reproducible LC-MS/MS and protein profiling are available, with millet stress-response applications, but annotation, low-abundance proteins and breeding-scale validation remain constraints.	(Ghatak et al., 2021)
Metabolomics	7	Established analytical platforms and millet stress-response studies exist, but environmental effects, metabolite annotation and standardization limit routine breeding deployment.	(Li et al., 2022b)
Phenomics	8	UAV, hyperspectral, thermal and digital phenotyping can be deployed under field conditions and integrated with genotype evaluation, although standardization and infrastructure remain challenges.	(Jadhav et al., 2024)
Epigenomics	5-6	Established analytical approaches exist, but millet-specific functional validation and evidence for stable breeding applications remain limited.	(Qadir et al., 2026)
Ionomics	6	Relevant for nutrient-use efficiency, ion homeostasis and salinity tolerance, but standardized databases and integration with breeding pipelines remain limited.	(Huang and Salt, 2016)
Lipidomics	5	Strong analytical potential for stress physiology, but millet-specific datasets and validated breeding applications are still limited.	(Pan et al., 2020)
Glycomics	4-5	Proof-of-concept and analytical capabilities exist, but technical complexity and limited millet reference resources constrain breeding application.	(Zhao et al., 2025)
Secretomics	4-5	Experimental applications are possible, but low-abundance proteins, contamination and limited millet-specific validation restrict translational maturity.	(Kasule et al., 2024)
Fluxomics	4-5	Provides mechanistic metabolic information, but isotope labelling, modeling requirements and limited millet breeding applications restrict readiness.	(Ghatak et al., 2025)
Redoxomics	4-5	Relevant to oxidative stress mechanisms, but transient signals, specialized instrumentation and limited millet validation constrain application.	(Supriya et al., 2025)
Rhizosphere Meta-omics	5	Increasingly useful for plant-microbiome interactions, but environmental variability and difficulty establishing causal relationships limit breeding deployment.	(Mattoo et al., 2021)
Microbiomics	6	Microbial diversity and PGPR-associated studies are established, but reproducibility across environments and functional validation remain challenges.	(Mazumder et al., 2025)
Single-cell & Spatial multi-omics	3–4	Strong proof-of-concept in plants, but limited millet applications, high cost, tissue-processing constraints, and lack of reference atlases restrict readiness.	(Yu et al., 2023)
Integrated multi-omics + AI	5–6	Demonstrated potential for genotype-to-phenotype prediction and predictive breeding, but data integration, standardized pipelines and independent field validation remain limiting factors.	(Li et al., 2025a)

## 3 Multi-stress biology: systems perspective for climate-resilient breeding

Millets are increasingly recognized as climate-resilient cereals because of their adaptation to marginal environments. Major millet crops include pearl millet (*Pennisetum glaucum*), finger millet (*Eleusine coracana*), foxtail millet (*Setaria italica*), proso millet (*Panicum miliaceum*), barnyard millet (*Echinochloa frumentacea*), little millet (*Panicum sumatrense*), kodo millet (*Paspalum scrobiculatum*), and sorghum (*Sorghum bicolor*) (Taylor and Kruger, 2018; Muthamilarasan and Prasad, 2021). Under field conditions, these crops may encounter drought, heat, salinity, flooding, and nutrient deficiency either sequentially or simultaneously. However, direct experimental evidence describing molecular responses of millet species to defined combinations of abiotic stresses remains comparatively limited. Therefore, in the following subsections, observations demonstrated specifically in millet crops are distinguished from regulatory mechanisms established more broadly in model plants and other crop species. General plant-stress mechanisms are discussed as biologically relevant frameworks for understanding potential multi-stress responses in millets, but they should not be interpreted as millet-specific mechanisms unless direct evidence is available (Zandalinas et al., 2021; Sithole et al., 2025). Accordingly, this section focuses on the systems-level mechanisms underlying combined abiotic stress responses, including heat-drought, heat-salinity, and flood-salinity interactions, as well as ROS-Ca^2+^ signaling, hormonal integration, and multi-omics-based network biology (**Figure 4**).

**Figure 4:**
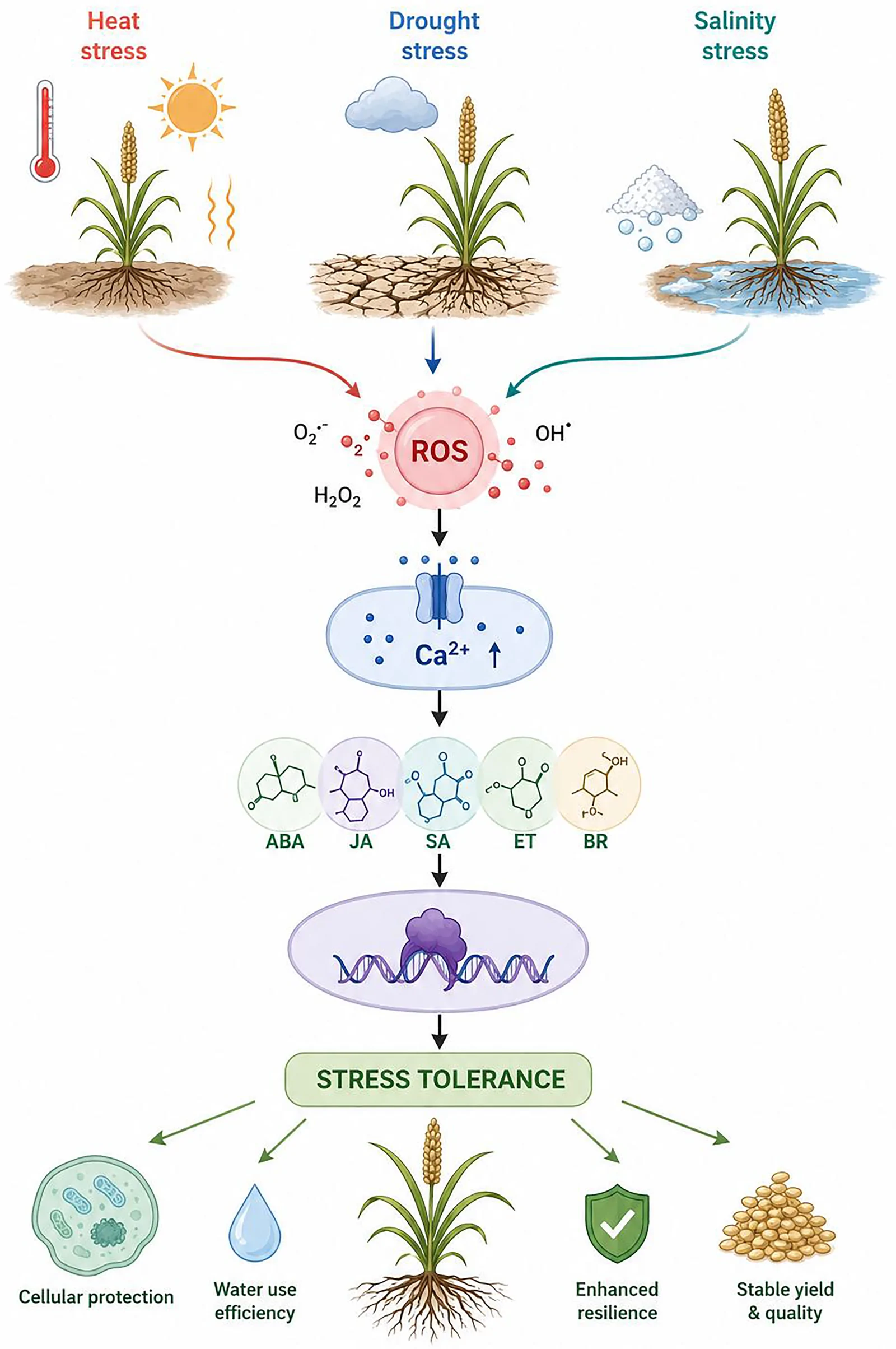
Convergent stress signaling network underlying abiotic stress tolerance in millet crops.

### 3.1 Heat-drought stress

Heat and drought frequently occur concurrently in semi-arid and arid environments and can substantially constrain millet productivity. Higher temperatures increase evapotranspiration and canopy water demand, impair pollen viability and photosynthetic performance, and can shorten the grain-filling period, whereas drought reduces water uptake, stomatal conductance and carbon assimilation (Singh et al., 2017). When imposed simultaneously, these stresses can produce stronger physiological and yield effects than either stress alone. The magnitude and nature of the interaction are not uniform and depend on genotype, stress intensity, timing, developmental stage and environmental conditions. In pearl millet, combined heat and water-deficit stress has been shown to cause stronger disruption of photosystem II electron transport than either stress applied individually, providing millet-specific evidence for an enhanced response under combined stress (Shanker et al., 2022). Similarly, foxtail millet genotypes exposed to combined heat and drought at anthesis showed greater yield reductions and physiological impairment than under individual stresses, although substantial genotypic variation in tolerance was observed (Gowsiga et al., 2024). Field studies in pearl millet further indicate that the timing and intensity of heat and water-deficit events contribute substantially to genotype × environment interactions. Thus, combined heat and drought should be considered as an interacting stress combination rather than assuming a universally synergistic response across millet genotypes and environments (Kapoor et al., 2026). Several adaptive responses associated with combined heat and drought stress have been reported in millets, including extensive root development, accumulation of compatible solutes such as proline and soluble sugars, enhanced antioxidant capacity, and regulation of heat-shock proteins (HSPs), dehydration-responsive element binding (DREB) transcription factors, and NAC, WRKY and MYB gene families.

### 3.2 Heat-salinity stress

In many dryland regions where irrigation is practiced, soil salinity is correlated with rising temperatures, creating simultaneous heat and salt stress conditions for millet crops. Osmotic stress and ionic toxicity are caused by high levels of Na^+^ and Cl^-^, while heat causes cellular membranes and protein complexes to become destabilized. These two effects act together to increase the production of reactive oxygen species (ROS), making the stability of chlorophyll, as well as the integrity of membranes, less stable, and causing reproductive development to be impaired (Zandalinas et al., 2021). Millets have efficient ion homeostasis systems including Na+/H+ antiporters (NHX), salt-overly sensitive (SOS) signaling components, high-affinity potassium transporters (HKT) and vacuolar sequestration systems, which maintain cell ion balance (Zhu, 2016). Concurrently, heat stress activates antioxidant enzymes such as superoxide dismutase, catalase, and ascorbate peroxidase, reducing oxidative damage (Hasanuzzaman et al., 2013; Mittler, 2017).

### 3.3 Flood-salinity stress

Millets are mainly grown under rainfed conditions, but coastal and low-lying environments may expose crops to overlapping waterlogging or flooding and salinity. Flooding primarily imposes oxygen limitation, whereas salinity causes osmotic stress and ionic toxicity; their co-occurrence can therefore impose distinct and potentially interacting constraints on root respiration, ion homeostasis, nutrient uptake, and root development (Sithole et al., 2025). Several mechanisms described for this stress combination are established primarily from studies of the individual stresses and should not be interpreted as having been demonstrated specifically in millet under simultaneous flood-salinity stress. For example, induction of anaerobic metabolism, including alcohol dehydrogenase and pyruvate decarboxylase, is a well-established response to flooding-induced hypoxia, whereas activation of SOS- and NHX-associated ion transport represents a recognized component of salinity adaptation (Terfa et al., 2025; Wang et al., 2025). These pathways provide plausible mechanistic components of the combined response, but their relative contribution and interaction under simultaneous flooding and salinity require direct experimental validation in millet. Evidence from broader plant studies indicates that additional signaling processes, including ethylene, Ca²^+^ signaling, and ROS homeostasis, may participate in responses to hypoxic saline conditions (Pan et al., 2020). Thus, rather than assuming that responses to flooding and salinity are simply additive, future millet studies should experimentally compare control, flooding, salinity, and combined flood-salinity treatments using time-resolved transcriptomics, ionomics, metabolomics, and physiological measurements. Such designs would help identify responses that are specific to the combined stress and distinguish them from pathways that are independently activated by either component stress.

### 3.4 Combined stress signaling networks

Plants do not experience multiple stresses in isolation but rather multiple environmental signals are integrated into multi-interconnected regulatory networks. Complex signaling pathways, such as those involving calcium ions (Ca²+), ROS, mitogen-activated protein kinase (MAPK) cascades, electrical signals, lipogenic messengers, and secondary metabolites, are involved in combined stresses (Suzuki et al., 2014; Zandalinas et al., 2021). These signaling elements work together to control transcriptional reprogramming, metabolic adaptation, and physiological adaptation. The calcium signatures generated are recognized by calcium-dependent protein kinases (CDPKs), calcineurin B-like proteins (CBLs), CBL-interacting protein kinases (CIPKs) and calmodulin-binding proteins, which in turn control stress-responsive transcription factors, including DREB, NAC, WRKY, MYB, and bZIP (Choi et al., 2014).

### 3.5 ROS-Ca²^+^ cross-talk during combined stress

Two highly interconnected second messengers, namely reactive oxygen species and calcium ions, coordinate early signaling of stress in plants. Plasma membrane NADPH oxidases (RBOHs), chloroplasts, mitochondria, and peroxisomes are the sites of rapid production of ROS that are stimulated by environmental stresses (Choi et al., 2014; Mittler, 2017). As a result of the oxidative burst, the cytosolic calcium levels generate a characteristic cytosolic calcium signature, with information for different combinations of stresses (Steinhorst and Kudla, 2013). On the other hand, calcium signaling can promote the generation of ROS by activating respiratory burst oxidase homologs, thereby establishing a ROS–Ca²^+^ signaling loop that amplifies ROS production (Gilroy et al., 2014). This reciprocal interaction regulates antioxidant defense systems, osmotic adjustment, programmed cell survival, and even transcriptional regulation (Mittler et al., 2022).

### 3.6 Hormonal integration under multiple stresses

Plant hormones act as master regulators that control physiological and molecular responses under combined environmental stresses. Abscisic acid (ABA) is the major regulator of drought and salinity response, acting to control stomatal closure, osmolyte accumulation and stress-responsive gene expression. The roles of ethylene are bifunctional in flooding tolerance; it is also involved in senescence regulation, while jasmonic acid (JA) and salicylic acid (SA) are involved in immune signaling and antioxidant defense (Waadt et al., 2022). Cytokinins are known to modulate source–sink relationships and delay leaf senescence, whereas auxins are known to control root architecture and adaptive root growth (Verma et al., 2016). Recently, multiple omics studies suggest that overall stress tolerance is regulated by a large-scale interaction between hormonal pathways, rather than any single one (Waadt et al., 2022).

### 3.7 Network biology and multi-omics integration

Combined stress responses are complex and require systems biology approaches that combine multiple molecular layers. Network biology integrates genomics, transcriptomics, proteomics, metabolomics, ionomics, phenomics and epigenomics to build gene regulatory networks, protein interaction networks, metabolic pathways and signaling modules involved in stress adaptation (Hasin et al., 2017; Jamil et al., 2020). The recent developments in the field of ML, graph-based biological networks, Bayesian inference, and AI have paved the way for predicting candidate regulatory genes, identifying hub transcription factors, and prioritizing genomic regions linked to complex stress tolerance (Varshney et al., 2020). Combination of GWAS, genomic selection, phenotyping, and multi-omics data supports the selection of indicators for breeding of climate-smart millet cultivars (Raza et al., 2024).

## 4 Genomics in millet crops

The advent of genomics has substantially advanced the investigation of abiotic stress adaptation in millet crops by enabling high-resolution characterization of genome organization, allelic diversity, and genomic regions associated with complex stress-related traits (Varshney et al., 2017; Muthamilarasan and Prasad, 2021). Early linkage mapping and QTL analyses provided the first genomic framework for dissecting traits such as drought response, stay-green phenotype, flowering time, grain yield and root architecture. Subsequent genome sequencing and high-throughput genotyping have expanded this framework by enabling GWAS, comparative genomics, genomic prediction and identification of candidate genes (**Table 3**) (Jadhav et al., 2024; Guo et al., 2025). Recently, chromosome-scale assemblies, long-read sequencing, pan-genomics, graph genomes and structural-variation analyses have increased the resolution at which adaptive genetic variation can be identified. These resources are particularly important for distinguishing conserved genomic regions from accession-specific alleles and structural variants that may contribute to environmental adaptation. Thus, current millet genomics is moving beyond reference genome characterization toward comprehensive discovery and prediction of functional genetic variation relevant to climate-resilient breeding.

**Table 3:** Genome resources and representative abiotic stress-responsive genes in major millet crops.

Millet species	Scientific name	Genome size (Mb)	Chromosome number (2n)	Reference genome availability	Abiotic stress-responsive genes/pathways	Reference
Pearl millet	*Pennisetum glaucum*	~1,760–1,790	14	Chromosome-scale reference genome available	*PgDREB2A, PgNAC, PgWRKY, PgHSP70, PgLEA, ABA signaling, ROS scavenging*	(Varshney et al., 2017)
Foxtail millet	*Setaria italica*	~395–420	18	High-quality chromosome-scale genome available	*SiDREB2, SiNAC, SiMYB, SiHKT1, SiP5CS, SiCDPK, heat shock proteins*	(Bennetzen et al., 2012)
Finger millet	*Eleusine coracana*	~1,450–1,600	36 (allotetraploid)	Chromosome-level reference genome available	*EcNAC, EcDREB, EcERF, EcLEA, antioxidant enzymes, calcium signaling*	(Hatakeyama et al., 2018)
Sorghum	*Sorghum bicolor*	~730–740	20	Well-annotated reference genome available	*DREB, NAC, Stg (Stay-green) loci*, *SbHSP, sSbWRKY, ABA-responsive genes*	(Vadez et al., 2013)
Proso millet	*Panicum miliaceum*	~920–930	36 (tetraploid)	Draft/chromosome-scale genome available	*PmDREB, PmNAC, HSPs, osmoprotectant biosynthesis, antioxidant pathways*	(Shi et al., 2019)
Little millet	*Panicum sumatrense*	~470–520	36	Draft genome available; improving assemblies	*Putative DREB, NAC, WRKY, ROS detoxification pathways, osmotic adjustment genes*	(Muthamilarasan and Prasad, 2021)
Kodo millet	*Paspalum scrobiculatum*	~850–900	40	Draft genome resources emerging	*Putative NAC, DREB, antioxidant defense genes, ion transporters*	(Muthamilarasan and Prasad, 2021)
Barnyard millet	*Echinochloa frumentacea*	~1,250–1,400	36	Genome resources emerging; draft assemblies available	*Heat shock proteins, antioxidant enzymes, NHX, osmolyte biosynthesis genes*	(Muthamilarasan and Prasad, 2021)
Browntop millet	*Urochloa ramosa*	~550–650†	36	Limited genomic resources; genome sequencing in progress	*Candidate stress-responsive transcription factors (DREB, NAC, MYB)* predicted from comparative genomics	(Muthamilarasan and Prasad, 2021)

### 4.1 Conventional genomics to stress biology

The initial investigations of the genome of millet crops were to develop a linkage map and locate QTLs for agronomic traits. These studies identified genomic regions associated with drought tolerance, stay-green phenotype, flowering time, grain yield, and root architecture, their relatively low mapping resolution limited the precise identification of causal genes (Vadez et al., 2011; Varshney et al., 2017).

### 4.2 Pan-genomics and genetic diversity of millets

Single reference genomes have been very useful for the progress of millet genomics, but resources based on individual reference genomes cannot fully capture the diversity of the millet genome. Pan-genomics has therefore become a very potent approach for the creation of comprehensive genomic resources consisting of conserved, core genes that are shared among accessions but also of dispensable genes that help accessions adapt to different environments (Golicz et al., 2016; He et al., 2023). Presence-Absence variations (PAVs), Copy number variations (CNVs), and novel genes of stress response that are not present in the conventional reference genomes have been identified in millet crops by using pan-genomic analyses (Varshney et al., 2021).

### 4.3 Graph genomes and structural variation

Traditional linear reference genomes may lead to reference bias and not capture complex genomic rearrangements. However, graph genome technology overcomes these limitations by stitching together multiple genome assemblies within a single genomic framework, so as to provide a more accurate representation of genetic diversity and structural variation. This method enhances sequence alignment, variant discovery and allele identification in various germplasm collections (Tao et al., 2019; Eizenga et al., 2020). Phenotypic diversity and environmental adaptation occur in large part due to structural variation such as insertions, deletions, inversions, duplications and translocations that influence gene dosage, gene regulation and gene organization (Alonge et al., 2020).

### 4.4 Haplotype-based genomics for precision breeding

Breeding strategies are increasingly employing haplotype analysis instead of individual SNP markers to capture favorable combinations of linked alleles inherited together in recent years. Highly diverse germplasm collections of millets contain superior haplotypes for drought tolerance, heat resistance, Nutrient-Use Efficiency (NUE) and grain yield, which can be used to improve breeding programs through haplotype-assisted selection (Bayer et al., 2020; Varshney et al., 2021). The use of haplotype information in conjunction with genomic prediction models also produced higher prediction accuracy for complex quantitative traits by accounting for the interactions between alleles and the linkage effects that are not accounted for by single marker prediction models.

### 4.5 Long-read sequencing revolution

Short-read sequencing has been hindered by a number of challenges, which have been partially addressed by third-generation sequencing platforms such as PacBio HiFi and Oxford Nanopore Technologies that produce highly contiguous chromosome-scale genome assemblies. Long-read sequencing can accurately characterize repetitive genomic elements, transposable elements, tandem duplications, and large structural variants, which have roles in the evolution of the genome and environmental adaptation (Logsdon et al., 2020). These technologies have also been used to facilitate full-length transcript annotation, identification of alternative splice forms, promoter identification, and stress-responsive regulatory sequence identification.

### 4.6 Genome editing for climate-resilient millets

Genome editing provides a promising route for translating candidate genes identified through genomics and multi-omics studies into improved millet germplasm. However, the level of experimental validation differs substantially among editing platforms and traits. CRISPR/Cas9 is currently the most established genome-editing technology in millets, particularly in foxtail millet (*Setaria italica*) (Liang et al., 2022). Targeted single- and multiplex-gene editing has been demonstrated for genes including *SiFMBP*, *SiDof4*, *SiBADH2*, *SiGBSS1*, and *SiIPK1*, with edited plants successfully recovered through Agrobacterium-mediated transformation (Liang et al., 2022). CRISPR/Cas9-mediated knockout of *SiZAT12* was shown to enhance drought tolerance in foxtail millet without an apparent yield penalty, providing direct evidence for genome-editing-mediated improvement of an abiotic-stress trait. Base editing has also been demonstrated experimentally in foxtail millet (Zhou et al., 2026). Cytosine and adenine base editors were used to target *SiALS* and *SiACC*, respectively, generating precise nucleotide substitutions and herbicide-tolerant plants. These results establish the technical feasibility of base editing in millet, although its application to drought, salinity, heat tolerance has not yet been directly demonstrated. In contrast, CRISPR/Cas12a, prime editing, and targeted epigenome editing remain prospective approaches for millet improvement (Ceasar, 2022). Accordingly, their proposed application to genes involved in ABA signaling, ion transport, osmotic adjustment, root architecture, flowering regulation, and ROS detoxification should be considered a future research direction rather than an experimentally validated outcome. Genome editing in millets is moving from proof-of-concept gene modification toward trait-oriented applications, but further work is required to improve transformation efficiency, broaden species and genotype coverage, assess off-target effects, and validate edited traits under field conditions. Integration of multi-omics-based candidate gene discovery with genome editing and multi-environment testing could facilitate the development of climate-resilient millet cultivars (Ceasar, 2022).

### 4.7 Current challenges in millet genomics

While the use of genomics in millet improvement has made great strides, there are still some hurdles to overcome. Additionally, several millets (orphan millet crops) are not represented in genomic databases and reference genomes of these crops are not available (Muthamilarasan and Prasad, 2021). In addition to the above, a few other challenges arose in genomic analysis, including polyploid genomes, high repetitive DNA content, structural variation, and limited functional validation of candidate genes (Bayer et al., 2020; Ho et al., 2020). Moreover, the majority of genomic studies have been conducted in response to a single abiotic stress in controlled conditions, whereas crops in the field are often subjected to several simultaneous abiotic stresses.

## 5 Transcriptomics in millet crops

Recent breakthroughs in genome sequencing have substantially expanded genomic resources for major millets, enabling the identification of quantitative trait loci (QTLs), genomic regions, and candidate genes associated with complex traits. High-quality chromosome-scale genome assemblies of foxtail millet (*Setaria italica*), pearl millet (*Pennisetum glaucum*), finger millet (*Eleusine coracana*), sorghum (*Sorghum bicolor*), and other millet species provide valuable resources for comparative genomics and predictive breeding (Hittalmani et al., 2017; Varshney et al., 2017). GWAS and linkage mapping have identified genomic regions associated with drought tolerance, flowering time, grain yield, root architecture, stay-green phenotype, and NUE. In pearl millet, major QTLs for terminal drought tolerance and grain yield stability have been identified, while genomic regions associated with transpiration efficiency and stay-green traits are being explored for drought-resistance breeding (Debieu et al., 2018; Parray et al., 2025). In sorghum, the classical stay-green loci (Stg1–Stg4) are among the best-validated genomic regions for post-flowering drought tolerance and have been used in marker-assisted breeding (Vadez et al., 2013). Conserved stress-responsive gene families, including *DREB, NAC, WRKY, MYB, bZIP, LEA, HSP, SOS1, NHX1, HKT1*, and *SnRK2*, have also been identified across millet crops, with roles in osmotic adjustment, ABA signaling, ion homeostasis, ROS detoxification, and cellular protection (Lata et al., 2010; Puranik et al., 2012). Complementing these genomic resources, transcriptomics provides a dynamic view of gene regulation under environmental stress. RNA sequencing (RNA-seq) enables the identification of differentially expressed genes, transcription factors, signaling pathways, and regulatory networks, while integration with QTL mapping, GWAS, and genomic selection can facilitate candidate-gene prioritization and molecular-marker development for climate-resilient breeding (Cramer et al., 2011; Crossa et al., 2017; Varshney et al., 2017).

### 5.1 Transcriptomics as a tool for stress-responsive gene discovery

Transcriptomics has emerged as one of the most impactful functional genomics methods to uncover the molecular causes of abiotic stress resistance in millets. RNA sequencing (RNA-seq) has been used to determine thousands of differentially expressed genes (DEGs), regulatory transcription factors, signaling pathways, and stress-responsive networks in response to drought stress, salinity stress, heat stress, and nutrient stress (Lata et al., 2010; Cramer et al., 2011). Integrating transcriptomic data with QTL mapping, GWAS, and genomic selection can help prioritize genomic regions, stress-responsive genes, and candidate markers, thereby potentially streamlining the process of candidate gene identification and marker development for climate resilience breeding (Crossa et al., 2017; Varshney et al., 2017). Despite the vast amount of transcriptomic information accumulated over the last decade, relatively few transcriptome-derived candidate genes have progressed to validated breeding applications, particularly in millets. Most transcriptomic studies have primarily contributed to stress-responsive gene discovery and candidate-gene prioritization, whereas their direct deployment in breeding has generally required integration with QTL/GWAS information, molecular markers, functional validation, and field evaluation (Sanku et al., 2024). For example, transcriptome-based analyses in rice have identified drought-responsive candidate genes within major drought-tolerance QTLs, including qDTY1.1, thereby providing targets for marker-assisted breeding and subsequent functional validation. Similarly, the Sub1A gene was identified as a major determinant of rice submergence tolerance through molecular and expression analyses and was subsequently deployed through marker-assisted selection in elite rice backgrounds (Muthu et al., 2020; Wu et al., 2021). These examples illustrate that transcriptomics can contribute substantially to breeding, but its greatest translational value is achieved when expression signatures are linked to stable genetic loci and validated at the phenotypic level. A major limitation is that transcript abundance does not always correlate with protein accumulation, enzymatic activity, or final phenotypic performance because of post-transcriptional regulation, translational control, and PTMs.

### 5.2 Experimental and technical limitations of current transcriptomic studies

A majority of investigations have been done under short-term, single stress conditions and a limited number of genotypes, while under field conditions, crops are exposed to varying combinations of drought, heat, salinity, nutrient deficiency and biotic stresses throughout their developmental life cycle (Zandalinas et al., 2021). This simplified experimental setup often neglects the genotype × environment interactions, as well as the temporal transcriptional responses, which limits the reproducibility and transferability of candidate genes from one breeding population to another. Additionally, sequencing depth, samples, developmental stage, bioinformatics pipelines, and thresholds for statistical significance cause challenges in cross-study comparison and meta-analysis (Conesa et al., 2016). Full-length transcriptomics has significantly enhanced transcriptome annotation, such as PacBio Iso-Seq and Oxford Nanopore sequencing, allowing accurate identification of full-length transcripts, alternative splicing forms, untranslated regions, and new genes (Zhao et al., 2019; Li et al., 2022a).

### 5.3 Emerging transcriptomic technologies: single-cell and spatial transcriptomics

Single-cell RNA sequencing (scRNA-seq) and spatial transcriptomics are revolutionary technologies that can uncover the unique stress responses in each cell type, which are not apparent in bulk transcriptome data. They have proved to be very successful in model plants like Arabidopsis and rice but have not been used to a great extent in millet crops (Ryu et al., 2021; Yu et al., 2023). Their implementation in non-model crops has been hindered by technical obstacles, including efficient isolation of protoplasts, the presence of rigid cell walls and the inability to dissociate tissues, low RNA levels, and high sequencing costs, as well as the lack of reference single-cell atlases (Shaw et al., 2021). Further, there are analytical challenges in cell clustering, trajectory inference and spatial data integration, with sophisticated computational approaches necessary for many plant breeding laboratories (Stuart and Satija, 2019). RNA-seq has greatly enhanced the resolution of gene identification in stress responsive millet crops. The transcriptomic analyses of various species of millets revealed large scale networks of differentially expressed genes, transcription factors, plant growth hormones, antioxidant systems and metabolic regulators involved in drought, salinity stress, and heat adaptation (Lata et al., 2010; Muthamilarasan and Prasad, 2021).

### 5.4 Temporal transcriptomics and translational challenges

Temporal transcriptomics has shown that the response to stress is multi-layered in terms of gene expression, with early-signaling genes showing different expression patterns from intermediate regulatory genes to the late-adaptive genes, as it progresses and recovers (Cramer et al., 2011). Many studies showed that they use single-point sampling time and thus miss transient regulatory events, which could be essential regulators of stress resilience. Experimental designs that incorporate time-series should thus be included in future studies to better capture the full picture of stress adaptation. While thousands of genes have been reported to be sensitive to stress, only a small fraction have been functionally validated using genome editing, mutant or transgenic methods.

### 5.5 Millet-specific transcriptomic insights and breeding applications

Recent RNA-seq studies have provided large amount of transcriptomic data for pearl millet, foxtail millet, finger millet and sorghum under drought, heat, salinity and nutrient stress. Transcriptome analyses of various plants have consistently shown upregulation of the families of transcription factors such as DREB, NAC, WRKY, ERF, MYB, and HSF, along with pathways of ABA-responsive signaling, calcium-dependent protein kinases, aquaporins, ROS-scavenging enzymes, and biosynthesis of osmoprotectants (Lata et al., 2010; Muthamilarasan and Prasad, 2015). In pearl millet, time-series RNA-seq analysis has revealed that dormancy is accompanied by rapid changes in transcriptomic profile, while transcriptome profiling in foxtail millet has revealed a large number of alternative splicing events across the entire genome and extensive networks of stress-induced regulatory events under drought stress (Qi et al., 2013).

## 6 Proteomics in millet crops

### 6.1 Conventional proteomics and stress-responsive protein discovery

Proteomics is a key functional omics platform which has come to the fore in bridging the gap between genotype and phenotype and can be used to reveal the full complement of proteins expressed at specific developmental stages or in response to environmental conditions (Ceasar et al., 2024). Proteomics has the advantage of offering direct information about protein abundance, PTMs, enzymatic activities, and protein–protein interactions, which represent important functional determinants contributing to stress adaptation in plants, while transcriptomics measures gene expression at the RNA level. Proteomics is essential to elucidate abiotic stress tolerance mechanisms in plants, as transcripts do not always reflect protein levels due to translational regulation, protein degradation, and PTMs (Kosová et al., 2018). Initially, traditional proteomic studies in millets were performed using two-dimensional gel electrophoresis (2-DE) followed by the identification of stress-responsive proteins by matrix-assisted laser desorption/ionization time-of-flight mass spectrometry (MALDI-TOF-MS) or liquid chromatography-tandem mass spectrometry (LC-MS/MS).

### 6.2 Quantitative proteomics and post-translational modifications

The application of recent developments in LC-MS/MS based quantitative proteomics has greatly improved the coverage of the plant proteome in species. Traditional gel-based studies found only a handful of stress-responsive proteins, while the new label-free quantitative proteomics (LFQ-MS), data-independent acquisition (DIA-MS) and SWATH-MS methods allow the quantification of large numbers of proteins and pathway analysis (Aebersold and Mann, 2016; Budzinski et al., 2019). Analysis also allows large-scale identification of phosphorylation sites, which can reveal information on the pathways of stress signaling by kinases. Under abiotic stress conditions, quantitative proteomics generally delivers information about proteins that are significantly regulated, such as those related to antioxidant defense, osmotic adjustment, hormone signaling and protein folding (Kosová et al., 2018). In addition to the abundance of protein, PTMs, such as phosphorylation, ubiquitination, acetylation, glycosylation and SUMOylation, provide rapid regulatory mechanisms that control protein function, stability, cellular localization, and protein interaction networks in response to environmental stress (Jiménez et al., 2025). In comparison, phosphorylation is one of the first molecular events to occur after the perception of stress.

### 6.3 Ubiquitin omics and protein interaction networks

Ubiquitination is an established post-translational regulatory mechanism involved in protein stability, signaling, and stress responses in plants. The ubiquitin–proteasome system (UPS), together with ubiquitin ligases and deubiquitinating enzymes, regulates the abundance and activity of proteins involved in transcriptional regulation, hormone signaling, and cellular stress responses (Stone, 2019). In millet species, the available evidence is currently more limited and is largely based on the identification, expression analysis, and functional characterization of individual ubiquitination-related genes rather than comprehensive ubiquitinomic profiling. For example, ubiquitination-related genes have been implicated in abiotic stress responses in foxtail millet, including RING-type E3 ubiquitin ligases and other components of the ubiquitination machinery. These findings provide evidence that ubiquitin-mediated regulation contributes to stress adaptation in millet, although the substrate-specific ubiquitination landscape and its dynamic changes under stress remain insufficiently characterized (Zhou et al., 2024). Therefore, large-scale ubiquitinomic approaches, including quantitative profiling of ubiquitinated proteins and integration with transcriptomic and proteomic datasets, represent a proposed next step for identifying additional ubiquitin regulators and their target proteins associated with stress tolerance and developmental plasticity in millet crops.

### 6.4 AI-integrated proteomics for predictive breeding

The combination of quantitative proteomics with genomics, transcriptomics, metabolomics, phenomics, and AI is revolutionizing the field of proteomics by turning it into a predictive tool that can be used in crop improvement. Integration of multi-omics data can help identify causal molecular pathways and thus enhance genomic prediction models and support precision breeding of climate-resilient cultivars (Hasin et al., 2017; Krassowski et al., 2020). However, multi-omics integration alone generally provides associative or mechanistic hypotheses rather than definitive evidence of causality; causal relationships require complementary approaches such as genetic perturbation, transgenic or genome-editing experiments, time-resolved analyses, and independent experimental validation. In finger millet, integrated transcriptomic and iTRAQ proteomic analyses identified thousands of differentially expressed proteins, with drought-responsive proteins enriched in carbohydrate metabolism, photosynthesis, redox processes, protein turnover and chaperone functions (Li et al., 2021). Sorghum proteomic studies similarly reported stress-induced changes in proteins involved in energy and carbohydrate metabolism, antioxidant defense, osmotic adjustment, protein synthesis and turnover, although the specific protein responses varied with genotype, tissue and experimental conditions (Jedmowski et al., 2014; Li et al., 2020). These findings indicate that proteomic responses to drought are conserved at the level of broad functional processes but can differ substantially among millet species, tissues, genotypes and stress regimes.

## 7 Metabolomics in millet crops

Metabolomics has proven to be an effective omics tool for elucidating the biochemical mechanisms of abiotic stress response and tolerance in millet crops. Metabolomics is a snapshot of the metabolites that represent the end products of cellular processes, different from genomics or transcriptomics, which reveal the genetic potential and gene expression dynamics (Dhawi, 2024). Metabolomics is useful as a link between genotype and phenotype as the metabolites are the products of integrated effects of gene expression, protein activity and environmental interactions.

### 7.1 Metabolomics platforms

The current metabolomics platforms based on GC–MS and LC–MS have greatly increased the coverage of metabolites that can be analyzed, beyond traditional biochemical approaches. Untargeted metabolomics provides full profiling of primary and secondary metabolites, while targeted metabolomics offers a high level of sensitivity in the quantification of specific metabolic pathways (Qiao et al., 2025). Advances in high-resolution mass spectrometry, including Orbitrap and time-of-flight platforms, have improved metabolite identification, pathway reconstruction, and comparative analysis of metabolites involved in stress-induced metabolic changes in plants (Schwender, 2011). Stable isotope- assisted fluxomics also allows the determination of carbon redistribution between interlinked metabolic pathways, giving dynamic insights into metabolic regulation in addition to static metabolite accumulation.

### 7.2 Spatial metabolomics

The recent progress in spatial metabolomics has greatly enhanced the potential for studying tissue-specific metabolic response in plants. The distribution of metabolites in plant tissue can be visualized simultaneously with spatial organization through mass spectrometry imaging, matrix-assisted laser desorption/ionization imaging, desorption electrospray ionization and laser ablation electrospray ionization (Boughton et al., 2016; Sturtevant et al., 2016). In this way, the localization of osmoprotectants, phytohormones, phenolic compounds and antioxidants in roots, vascular tissues, guard cells, developing grains, and reproductive organs under abiotic stress conditions is achieved. Spatial metabolomics can thus offer important insights into tissue-specific adaptation mechanisms that are not captured by traditional bulk metabolite extraction methods.

### 7.3 Fluxomics

Another potential solution is “fluxomics,” a method that analyses the flow of metabolites in and out of tightly coupled metabolic networks in addition to metabolite levels. During stress adaptation, Fluxomics can deliver quantitative flux data for carbon and nitrogen partitioning through glycolysis, the tricarboxylic acid (TCA) cycle, the pentose phosphate pathway, photorespiration, amino acid biosynthesis, and secondary metabolic pathways (Fernie and Morgan, 2013; Cui et al., 2023). Metabolic flux redistribution in crop plants defines the ability of tolerant genotypes to reroute assimilated carbon to osmolyte biosynthesis, antioxidant production and energy conservation under extended water deprivation and/or heat stress.

### 7.4 Lipidomics

The field of lipidomics has emerged as a powerful tool to gain a deeper insight into how membrane remodeling occurs in response to environmental stress. Lipidomic analysis provides information on the phospholipids and galactolipids, sphingolipids, glycerolipids, oxylipins and signaling lipids involved in membrane stability, cellular signaling and stress adaptation (Shiva et al., 2018). Plant genotypes tolerant to drought and heat stress, ensure the integrity of the membrane by controlling the composition of the lipids, which regulates the abundance of phosphatidylcholine, phosphatidylethanolamine, phosphatidylglycerol, and galactolipid, thereby maintaining the efficiency of photosynthesis and the stability of the cells under drought and heat stress (Ren et al., 2025).

### 7.5 Volatilomics

Volatilomics, a new field of research, has been developed to profile the volatile organic compounds that plants release as a result of responding to an environmental challenge. Metabolites that are volatile, such as terpenoids, green leaf volatiles, aldehydes, alcohols, esters, and methylated compounds, serve to signal and act as mediators in oxidative stress reduction, intercellular communication and benefits for beneficial microorganisms, among others (Dudareva et al., 2013; Scala et al., 2013).

### 7.6 Stable isotope tracing

Stable isotope tracing has also been applied to further enhance metabolomic studies, allowing for an estimation of the activity of metabolic pathways. Metabolic substrates can be labeled with isotopes (^13^C, ^15^N, ^2^H, ^18^O) to track the assimilation of carbon, allocation of nitrogen, biosynthesis of amino acids, photorespiration, and respiratory metabolism during stress (Wiggenhauser et al., 2022). Metabolomics in combination with stable isotopes allows for the characterization of active metabolic fluxes within a genotype, in order to gain mechanistic insights into the resource allocation strategies of stress-tolerant millet genotypes (Allen et al., 2009; Schwender, 2011). Combining isotope tracing with fluxomics can identify central nodes in the regulatory network that underlie stress adaptation and efficient use of nutrients.

### 7.7 Metabolomic biomarkers

Abiotic stress tolerance has been studied in millets and characteristic biochemical signatures have been identified using metabolomic analyses. The drought-tolerant pearl millet also has higher levels of proline, raffinose, trehalose, γ-aminobutyric acid (GABA), flavonoids and phenolic compounds, while the finger millet showed higher levels of calcium-associated metabolites and antioxidant molecules, which are involved not only in stress adaptation but also in nutritional qualities (Saleem et al., 2021). Under water deficit and heat stress, foxtail millet exhibits significant metabolic re-programming of amino acids, sugars, and lipid-derived metabolites.

## 8 Phenomics in millet crops

Phenomics has become a revolutionary science for crop improvement and provides the ability to measure in large-scale and quantitatively, plant traits during growth and development under a variety of environments. Compared to traditional phenotyping, which is labor-intensive, subjective and captures only a few traits, phenomics is creating opportunities to rapidly, non-destructively and accurately record high-dimensional phenotypic information using advanced imaging technologies, sensor platforms, robotics and AI (Furbank and Tester, 2011; Araus and Cairns, 2014). In millets, with complex abiotic stress responses that rely on several interacting genetic and physiological mechanisms, high-throughput phenotyping is a necessary link between the genomic variation and agronomic performance (Cobb et al., 2013; Tardieu et al., 2017). The growing importance of millets as climate-smart cereals is due to their inherent drought, heat and nutrient-limited tolerance. Despite all of the progress that has been made in the development of genomic resources, superior stress-resilient genotypes are limited by phenotyping bottlenecks (Reynolds et al., 2021).

### 8.1 UAV-based field phenomics

Unmanned Aerial Vehicles (UAVs) with multisensory imaging systems have transformed the phenotyping of millet in the field. UAV platforms also provide the ability to quickly assess large breeding populations, with reduced labor requirements and environmental disturbance (Araus and Cairns, 2014; Sankaran et al., 2015). Canopy architecture, plant height, flowering synchrony, biomass accumulation, lodging resistance and stress progression over the growth season are measurable using high-resolution aerial imagery (Li et al., 2019; Yang et al., 2021). Temporal monitoring of crop development is possible, as repeat UAV surveys provide a description of development over time, not just at one point, allowing breeders to describe growth dynamics. The use of UAV platforms to collect time-series phenotypic data has been a major improvement in the detection of drought-adaptive growth and genotype-specific responses to changing environmental conditions.

### 8.2 RGB imaging for morphological trait analysis

The simplicity, low cost and versatility of ground-based and aerial RGB imaging make it one of the most widely used phenotyping methods. When measuring the morphological traits of grain sorghum via high-resolution RGB image analysis, many parameters can be automatically estimated: canopy cover, plant height, number of tillers, leaf area, panicle architecture, lodging severity, and biomass accumulation (Fahlgren et al., 2015). In recent years, deep-learning algorithms that segment and detect objects in images have been developed, which have made it possible to automatically extract these traits from images (Ubbens et al., 2018). Models trained by ML using RGB data can correctly identify each plant, estimate the amount of canopy and monitor crop growth across the growing season.

### 8.3 Hyperspectral imaging for physiological phenotyping

Hyperspectral imaging is, in recent years, one of the most useful non-destructive methods to detect physiological changes before the onset of visible stress responses. In contrast to conventional RGB imaging, hyperspectral sensors measure reflectance data in hundreds of narrow spectral bands, thus allowing detailed characterization of the biochemical composition and physiological state of plants (Li et al., 2014; Mahlein, 2016). For millet crops, the hyperspectral imaging technique has been successfully used to estimate the chlorophyll concentration, leaf nitrogen content, water status, photosynthetic efficiency, pigment composition, and early responses to stress (Araus and Cairns, 2014; Mahlein, 2016). Hyperspectral data can be used to generate spectral vegetation indices that are useful for fast discrimination of tolerant and susceptible genotypes in drought, heat and nutrient-deficient environments (Fiorani and Schurr, 2013).

### 8.4 Thermal imaging for early stress detection

Indirect assessment of plant water status can be made using thermal infrared imaging to measure canopy temperature (transpiration efficiency and stomatal conductance). During drought or heat stress, the canopy temperature will rise, even before the plants show wilt symptoms, due to lower transpiration (Costa et al., 2013). Estimation of canopy temperature depression, stomatal behavior and transpiration efficiency has emerged as an effective way to identify drought-tolerant millet genotypes using a thermal phenotyping approach (Araus and Cairns, 2014; Furbank et al., 2019). The combination of physiological and structural information is highly beneficial for stress detection by integrating thermal, hyperspectral and RGB datasets, which produces greater accuracy.

### 8.5 LiDAR-based three-dimensional phenotyping

A method called Light Detection and Ranging (LiDAR) can generate very accurate 3D models of the plant architecture based on the distance from a laser pulse to plant surfaces. Using LiDAR-derived point clouds, complex plant height, canopy volume, leaf angle distribution, stem architecture and biomass accumulation can be estimated accurately without destructive sampling (Tattaris et al., 2016; Paulus, 2019). LiDAR has many benefits in quantifying structural properties related to lodging resistance, biomass production and radiation interception, which are relevant to millet breeding (Araus and Cairns, 2014). A 3D canopy reconstruction also allows for better model light interception efficiency and crop growth under varying environmental conditions.

### 8.6 Root phenomics

While above-ground attributes have long been at the core of breeding programs for millets, root architecture is gaining in significance as a key factor affecting drought adaptation and nutrient uptake in millet crops. The goal of root phenomics is to measure the rooting system, which includes rooting depth, root angle, lateral branching, root density, and root biomass (Rogers and Benfey, 2015; Lynch, 2019). Studies can significantly benefit from the use of non-destructive imaging techniques, like X-ray computed tomography, magnetic resonance imaging, rhizotrons, minirhizotrons, and transparent growth systems (Julkowska et al., 2017; Atkinson et al., 2019). Furthermore, root image analysis using ML algorithms can be used to automatically extract complex parameters of the root architecture with a high level of precision.

### 8.7 Digital phenotyping and artificial intelligence

Phenomics is becoming a data-centric discipline, and the advent of digital agriculture has led to the integration of imaging platforms, environmental sensors, IoT devices, cloud computing, and AI-related analytics (Furbank and Tester, 2011). Digital phenotyping involves integrating ongoing data collection from sensors with automated image analysis to produce detailed field-grown plant phenotyping data. The use of multidimensional omics data has greatly enhanced genomic prediction and phenotype forecasting, and has been greatly aided by the use of AI-based approaches (Crossa et al., 2017; van Dijk et al., 2021). Large-scale image datasets (from 100 to 10,000) are very suitable for automated phenotyping and stress classification using machine-learning models such as Random Forest, DNNs, and Convolutional Neural Networks (CNNs) (Ubbens et al., 2018).

### 8.8 Millet field phenomics

Large-scale UAV-based phenotyping has been adopted in pearl millet and sorghum breeding programs to assess canopy temperature, stay-green traits, biomass accumulation, flowering phenology and drought tolerance for large breeding populations (Araus and Cairns, 2014; Haghighattalab et al., 2016). High-throughput phenotyping combined with GWAS has led to the discovery of genomic regions related to canopy temperature depression, transpiration efficiency, biomass production and drought adaptation traits (Kholová et al., 2012; Vadez et al., 2013). Hyperspectral imaging, RGB imaging and thermal cameras are being used in an increasing number of Foxtail millet and Finger millet phenotyping studies to detect drought stress at early stages and deep-rooting phenomics to identify genotypes adapted to water-limited environments.

## 9 Epigenomics in millet crops

### 9.1 Epigenetic regulation of abiotic stress responses

One of the important concepts in plant epigenomics is stress memory, in which a previous exposure to abiotic stress can leave molecular changes that persist after stress removal and influence the plant’s response to a subsequent stress episode. These memory effects may involve changes in DNA methylation, histone modifications, chromatin accessibility, and stress-responsive gene expression, and can contribute to a faster or stronger response upon re-exposure (Qadir et al., 2026). Importantly, stress memory within an individual should not be equated with transgenerational inheritance. Persistence of an epigenetic state through mitotic cell divisions within the same plant or vegetative lineage represents within-generation memory, whereas transmission through meiosis to progeny constitutes heritable or transgenerational epigenetic inheritance and requires evidence that the altered state persists in subsequent generations. Thus, although stress-induced epigenetic modifications may contribute to both phenomena, their stability, mechanisms, and biological significance should be considered separately (Kim et al., 2015; Ueda and Seki, 2020).

### 9.2 Stress memory and transgenerational epigenetic inheritance

Stress memory and transgenerational epigenetic inheritance represent related but distinct phenomena. Stress memory refers primarily to the persistence of stress-associated molecular states within an individual plant, including through mitotic cell divisions, such that a subsequent stress exposure can elicit an altered response. In contrast, transgenerational epigenetic inheritance refers to the transmission of an epigenetic state or associated phenotype through reproductive generations (Wang et al., 2024). Demonstrating such inheritance requires evidence that the phenotype or epigenetic mark persists in progeny after the original environmental stress has been removed and, where appropriate, after controlling for parental and environmental carry-over effects. While DNA methylation, histone modifications, and chromatin-associated mechanisms have been implicated in stress memory and, in some plant systems, in heritable stress responses, the stability and frequency of true transgenerational inheritance remain variable and context-dependent. In particular, evidence for stable transgenerational epigenetic inheritance in millet crops remains limited. Therefore, epigenetic memory should not be assumed to be transmitted across generations without direct multigenerational evidence (Herman and Sultan, 2011; Springer and Schmitz, 2017; Wang et al., 2024).

### 9.3 Chromatin accessibility profiling and emerging epigenomic technologies

Assay for Transposase-Accessible Chromatin using sequencing (ATAC-seq) has increasingly been applied to millet crops, providing direct evidence of stress- and tissue-associated chromatin accessibility. In foxtail millet (*Setaria italica*), recent ATAC-seq and RNA-seq analyses identified substantial changes in chromatin accessibility during drought recovery and recurrent drought stress, with enrichment of AP2/ERF transcription-factor motifs in accessible regions (Li et al., 2025b). Similarly, integrated epigenomic profiling of pearl millet (*Pennisetum glaucum*) using ATAC-seq, DNA methylation, Chromatin Immunoprecipitation followed by sequencing (ChIP-seq) and RNA-seq revealed tissue-specific accessible chromatin regions enriched for stress-responsive transcription-factor motifs, including NAC and WRKY (Luo et al., 2025). These findings indicate that ATAC-seq is moving beyond a prospective application in millets toward an emerging experimental approach for resolving regulatory elements associated with stress adaptation and developmental processes.

### 9.4 Multi-omics integration in millet epigenomics

Epigenomic data integration with genomics, transcriptomics, proteomics, metabolomics and phenomics is building a systems-level understanding of stress adaptation in millet crops (Varshney et al., 2020; Yang et al., 2021). The multi-omics analyses can be used to separate the causal epigenetic regulators from downstream stress-responsive genes, identify stable molecular markers, and discover regulatory hubs that control multiple abiotic stress pathways at once (Ueda and Seki, 2020). Data combined with GWAS, genomic prediction, and AI-enabled network analysis provide unprecedented opportunities to identify functional regulatory elements and to enhance breeding efficiency (Crossa et al., 2017; van Dijk et al., 2021). Although these advances have been made, there are still several hurdles to overcome to have epigenomic information readily incorporated into breeding programs. Stress-induced epigenetic changes are stable over time, tissue-specific chromatin dynamics remain yet to be explored, environmental changes affect epigenetic states and adaptive epialleles are heritable under field conditions; these are important aspects that need to be addressed (Lämke and Bäurle, 2017; Springer and Schmitz, 2017).

## 10 Ionomics in millet crops

The field of Ionomics, the integrated study of the elemental composition (ionome) of an organism, has now become a valuable systems biology tool to help understand mineral nutrient acquisition, transport, accumulation and homeostasis in a variety of environments. Ionomics also has the advantage of measuring many different macro- and micronutrients at the same time and will help researchers to explore complex relationships between mineral elements and the genes that regulate them (Salt et al., 2008). For crops naturally grown in harsh conditions and low nutrient soils, as in case for millets, ionomics can be used to gain insight into the physiological and molecular basis of stress tolerance, NUE, and biofortification potential (Baxter et al., 2012; Muthamilarasan and Prasad, 2021). Several millet species and genotypes have demonstrated substantial tolerance or adaptation to abiotic stresses such as drought, heat, and salinity, while some also perform relatively well under nutrient-limited conditions. However, the magnitude and mechanisms of stress tolerance vary among species, genotypes, developmental stages, and environmental conditions. For example, stress-responsive mechanisms involving ion homeostasis, osmotic adjustment, antioxidant defense, and nutrient acquisition have been reported in species such as pearl millet (*Pennisetum glaucum*), finger millet (*Eleusine coracana*), and foxtail millet (*Setaria italica*).

### 10.1 Ionomics for improving nutrient-use efficiency in millets

There is a high breeding priority regarding NUE, as the excessive use of fertilizer leads to high production costs and environmental pollution. Millets have proven to be superior models of sustainable agriculture due to their nutrient acquisition and utilization efficiencies (Muthamilarasan and Prasad, 2021). Ionomic analysis can be used to identify genotypes that can sustain optimal nutrient uptake and internal nutrient balance under nutrient-deficient conditions. Changes in the accumulation of these elements can be correlated to genes involved in nutrient transport, re-mobilization and storage, leading to the possibility of marker-assisted selection and genomic selection for higher NUE (Salt et al., 2008; Baxter et al., 2012).

### 10.2 Element interaction networks under abiotic stress

Coordinated interactions between several mineral elements are as important as individual ions for plant adaptation to abiotic stress. Element interaction networks refer to interactions between nutrients that work synergistically and antagonistically to ensure homeostasis of cells in conditions of stress (Salt et al., 2008). For instance, high K^+^/Na^+^ is a basic requirement of salinity tolerance, and Ca²^+^ is a structural molecule and an important secondary messenger in stress-responsive signaling pathways (Munns and Tester, 2008; Deinlein et al., 2014). Similarly, the relationships between Mg, Fe, Mn, and P affect photosynthesis, enzyme activation, antioxidant defense, and metabolic regulation. By combining network-based ionomic analyses, researchers can identify co-regulated elemental modules and discover the regulatory relationships not previously known that contribute to stress adaptation (Baxter et al., 2012).

### 10.3 Ion transport modeling and systems-level understanding

The recent progress in computational biology has allowed the development of models of ion transport that simulate nutrient uptake, long-distance transport, compartmentalization, and redistribution in various plant tissues. These models incorporate transporter kinetics, membrane transport processes, environmental factors and physiological parameters to simulate nutrient dynamics under varying environmental conditions (Salt et al., 2008; Baxter et al., 2012). In the case of millet crops, modeling of ion transport can be used to better understand the responses of drought and salinity stress on root architecture-mediated nutrient acquisition, xylem–phloem transport efficiency, vacuolar sequestration, and ionic homeostasis.

### 10.4 AI-based ionomic prediction for precision breeding

The increasing availability of high-throughput elemental profiling provides opportunities to apply AI and machine-learning approaches to ionomic datasets. Supervised algorithms such as Random Forest, Support Vector Machine, Extreme Gradient Boosting (XGBoost), and DNNs can potentially be used to classify stress responses and predict nutrient-related traits from elemental profiles (Yan and Wang, 2023). However, their performance is strongly dependent on the size, quality, and representativeness of the training datasets, and direct validation of ionomics-based ML models for stressed-genotype classification and NUE prediction remains limited in millet crops. Recent millet studies demonstrate the broader potential of ML for genotype and trait prediction, including metabolomics-based prediction in pearl millet, but these should not be considered equivalent to validated ionomic prediction of NUE (Singhal et al., 2025). Therefore, millet-specific datasets combining elemental profiles with replicated stress and NUE phenotypes, together with independent validation across environments and genotypes, are required before such models can be considered reliable for precision breeding. Unsupervised approaches may additionally help identify clusters of ions and elemental signatures associated with stress, whereas graph-based approaches offer a framework for exploring complex nutrient-interaction networks (Salt et al., 2008).

## 11 Emerging omics in millet crops

The emergence of omics technologies has revolutionized plant breeding from the one-dimensional molecular approach to systems biology. The evolution and development of several new disciplines of the omics sciences have shown considerable progress in understanding abiotic stress tolerance in millet crops, and some more recent areas of omics sciences are beginning to give deeper insight into plant physiology, metabolism, and plant-microbe interactions (Banshidhar et al., 2023; Kumar et al., 2026).

### 11.1 Lipidomics

Lipidomics is the study of the entire landscape of lipids and their changes in response to environmental stress in the cell. In addition to their structural functions in biological membranes, lipids are important signaling molecules that play a role in maintaining the integrity of biological membranes, energy storage, and stress perception (Hou et al., 2016). When abiotic stresses like heat, salinity, and drought occur, they cause extensive remodeling of membrane phospholipids, galactolipids, sphingolipids, and oxylipins, maintaining fluidity and cellular homeostasis (Narayanan et al., 2016). While the number of studies is relatively limited in comparison to the major cereals, emerging evidence shows that tolerant millets have differential accumulation of lipids, such as phosphatidylcholine, phosphatidylethanolamine, phosphatidylglycerol, and galactolipids during drought and heat stress. Lipids signal through calcium signaling, ROS and ABA pathways in response to stress (Hou et al., 2016).

### 11.2 Glycomics

Glycomics is an approach to the global study of glycans, glycoproteins, glycolipids, and polysaccharides of the cell walls involved in the regulation of plant growth, development, and environment. One of the most common PTMs that affect the stability, receptor signaling and stress-induced metabolic pathways of proteins is glycosylation (Li et al., 2021). Millets have changes in cell wall polysaccharides, hemicellulose, pectin and arabinoxylans, which promote increased resistance to mechanical damage, osmotic adjustment and water retention in response to drought and salinity stress (Burton et al., 2010). The biosynthesis of these complex carbohydrates is controlled by glycosyltransferases, and they are involved in cell wall plasticity in stress adaptation (Mohnen, 2008). Advanced glycomics techniques, such as mass spectrometry, capillary electrophoresis, glycan microarrays and lectin-based glycan profiling can be used to gain insights into stress tolerance-related structural changes.

### 11.3 Secretomics

Secretomics is the total set of proteins secreted outside the cell, in the apoplast or rhizosphere. Cell-to-cell communication, pathogen resistance, cell wall remodeling, nutrient uptake and environmental sensing are all important roles of the plant secretome (Agrawal et al., 2010). The secretome is significantly changed by abiotic stress, which triggers the production of antioxidant enzymes, hydrolytic enzymes, expansins, peroxidases, heat shock proteins and signaling peptides (Alexandersson et al., 2013). Secreted proteins are involved in maintaining cell wall integrity, scavenging of reactive oxygen species and the regulation of root architecture in millet under drought and nutrient-deficient conditions.

### 11.4 Fluxomics

In contrast to traditional metabolomics, which reflects the level of metabolites when they are in a steady state, fluxomics reflects the dynamic flow of metabolites through interconnected biochemical pathways (Schwender, 2011). Stable isotope labeling with ^13^C, ^15^N or ^2^H, together with GC-MS, LC-MS and nuclear magnetic resonance (NMR), can be used to measure metabolic fluxes accurately under varying environmental conditions (Allen et al., 2009). Under abiotic stress, millet plants redirect carbon and nitrogen resources for osmo-protectant biosynthesis, antioxidant metabolism and energy conservation. Fluxomics analyses can be used to detect bottlenecks in glycolysis, the tricarboxylic acid (TCA) cycle, photorespiration, amino acid biosynthesis and secondary metabolism (Obata and Fernie, 2012).

### 11.5 Redoxomics

Redoxomics involves the study of reversible oxidative modification of proteins, metabolites, and nucleic acids that mediate cellular oxidation–reduction processes that regulate signal transduction and stress responses. The production of ROS in abiotic stress conditions can play a role as detrimental agents as well as signaling molecules that regulate stress-related gene expression. Millet crops deploy diverse antioxidant defense components, including superoxide dismutase, catalase, ascorbate peroxidase, glutathione reductase, peroxiredoxins, and thioredoxins, which contribute to cellular redox homeostasis (Mittler, 2017). The identification of reversible protein modifications like S-nitrosylation, glutathionylation, sulfenylation and disulfide bond formation is possible in recent years due to improved techniques of redox proteomics, fluorescent probes for redox modifications and quantitative mass spectrometry analysis.

### 11.6 Rhizosphere meta-omics

The rhizosphere is one of the most biologically dynamic interfaces of plants and soil microorganisms. Rhizosphere meta-omics combines metagenomics, meta-transcriptomics, metaproteomics, and metabolomics to provide a comprehensive view of microbial community structure and inter-microbial interactions in the rhizosphere of plants. Under marginal conditions, beneficial rhizosphere microorganisms play a significant role in the uptake of nutrients, drought tolerance, phosphorus solubilization, biological nitrogen fixation, phytohormone production and stress alleviation, all of which are important for millet growth (Bulgarelli et al., 2013).

### 11.7 Microbiomics

Microbiomics is the study of microbial communities, in particular their composition, diversity, functional potential, and dynamics within various plant parts and tissues. Beneficial bacteria and fungi affect nutrient cycling, hormone signaling, immune priming, and abiotic stress tolerance (Mazumder et al., 2025). Recent studies suggest that drought-tolerant millet (DTM) genotypes may be associated with distinct microbial community compositions, including plant growth-promoting rhizobacteria (PGPR), arbuscular mycorrhizal fungi, and endophytic microorganisms, with potential roles in processes related to water acquisition, antioxidant activity, and nutrient uptake (Chandra et al., 2020). However, the extent to which these microbial associations contribute directly to drought tolerance remains to be established through functional and field-based validation. The ability to profile microbial communities using high-throughput sequencing technologies such as 16S rRNA gene sequencing, ITS sequencing, shotgun metagenomics, and long-read sequencing has greatly advanced our understanding of microbial communities (Niu et al., 2018).

## 12 Single-cell and spatial multi-omics in millet crops

The ability to characterize the molecular content of individual cells and tissues with unprecedented resolution has transformed plant systems biology in recent years, with the development of single-cell and spatial multi-omics (Baysoy et al., 2023). Single-cell technologies provide cell-type-specific gene expression, chromatin accessibility, metabolic activity, and regulatory networks that govern plant growth and stress adaptation, and offer a powerful alternative to conventional bulk omics approaches, which average signals across heterogeneous cell populations.

### 12.1 Single-cell RNA sequencing

scRNA-seq has become one of the most revolutionary methods to understand plant cellular diversity in the context of development and environmental response. scRNA-seq can profile transcriptomes at the single-cell level and allow one to identify rare cell populations, developmental trajectories, and stress-responsive regulatory networks, which were not identified by bulk transcriptomic analyses (Shaw et al., 2021). For cereals, scRNA-seq has been used to reveal different transcriptional programs in the root epidermis, cortex, endodermis, pericycle, vascular tissues, and guard cells in response to drought, salinity and heat stress. While most species of millet remain poorly represented in scRNA-seq datasets, there is a window of opportunity for generating single-cell transcriptomic atlases for foxtail millet, pearl millet, finger millet, proso millet, and sorghum.

### 12.2 Spatial transcriptomics

The scRNA-seq approach yields high-resolution transcription information but the dissociation of cells removes the spatial context. Spatial transcriptomics has now been developed, in which both the tissue architecture and the gene expression of an intact organ is preserved. The technology allows researchers to see the location of the genes that react to stress in a particular tissue, and thus the molecular response varies with location during environmental stress. Spatial transcriptomics promises to provide tremendous opportunities for studying the underlying gene expression changes that occur across the plant in different tissues in response to drought, salinity, heat, and nutrient deficiency stresses in millet crops (Shao et al., 2026).

### 12.3 Spatial metabolomics

Metabolites are the end products of cellular regulatory process and hence they offer the best molecular connection to plant phenotype. However, traditional metabolomic analysis generally involves homogenization of tissues, which obscures local metabolic responses. To assess the spatial profile of primary and secondary metabolites in whole tissues, spatial metabolomics is a combination of mass spectrometry imaging and metabolite profiling (Boughton et al., 2016) Spatial metabolomics has not been widely used in millet research and will have great potential to elucidate the accumulation of osmoprotectants, antioxidants, phytohormones, flavonoids, amino acids and compatible solutes at a specific site during abiotic stress. Metabolite mapping in root, vascular bundle, reproductive tissues and developing grains will be useful to gain insights into tissue-specific metabolic reprogramming at the heart of stress tolerance.

### 12.4 Single-cell epigenomics and ATAC-seq

The accessibility of chromatin and epigenetic regulation have a profound effect on the expression of genes. scATAC-seq is a single-cell Assay for Transposase-Accessible Chromatin sequencing that allows for the genome-wide identification of open chromatin regions at cellular resolution, which will provide a means to identify regulatory elements regulating stress-responsive transcription (Buenrostro et al., 2015). The application of scATAC-seq has been mainly in model plants, but when applied to millet, it will facilitate the identification of abiotic stress adaptation-associated cell-specific regulatory elements, transcription factor binding sites, enhancer regions, and chromatin dynamics. By integrating scATAC-seq with scRNA-seq, one can obtain direct links between chromatin accessibility and gene expression, enabling reconstruction of networks of genes that are regulated to respond to stress.

### 12.5 Cell-type-specific stress responses

The cells’ reactions to environmental stress are highly cell type specific. The root epidermal cells control water and nutrient uptake, the cortical cells are involved in osmotic adjustment, the endodermal cells in ion homeostasis, the vascular cells in long-distance signaling and the guard cells in stomatal conductance and transpiration (Jin et al., 2025). This makes it difficult to detect important regulatory events within specialized cell populations using bulk omics approaches. Single-cell and spatial multi-omics offer unparalleled insights into the responses of individual cell populations in drought, salinity, heat, nutrient deficiency, and in combination. These strategies would help to determine distinct root system, hydraulic conductivity, ion transport, photosynthetic efficiency, reproductive development and grain filling stress regulatory networks in millets.

## 13 Data integration and systems biology in millet crops

### 13.1 Systems biology for multi-omics integration

With the tremendous development in high-throughput omics technologies, a huge amount of genomic, transcriptomic, proteomic, metabolomic, phenomic, epigenomic, and ionomic data have been generated in millet crops. Each omics platform offers unique information about specific biological processes, but each dataset only captures a small part of the complexity of abiotic stress tolerance. Hence, systems biology involving the integration of multiple omics layers has become a promising approach to understand the intricate networks of regulation underlying stress adaptation and speed up the process of improvement of climate-smart millets (Hasin et al., 2017). Multi-layer data integration allows for simultaneous analysis of genetic variation, gene expression, protein abundance, metabolite accumulation, elemental composition and phenotypic performance. Combining these complementary datasets has enabled the identification of key regulatory genes, signaling pathways, metabolic hubs and molecular indicators of drought, salinity, heat and nutrient stress in species of millets (Yang et al., 2021).

### 13.2 Network inference and causal modeling for stress regulatory networks

Network inference, the reconstruction of biological interaction or association networks from large-scale omics data, is a fundamental component of systems biology. Gene co-expression networks, protein–protein interaction networks, metabolic association networks, transcription factor regulatory networks, and signaling networks can be used to identify co-regulated modules, highly connected hub genes, and candidate relationships associated with stress responses. For example, integration of transcriptomic and metabolomic data in millets can reveal coordinated patterns involving osmolyte biosynthesis, antioxidant metabolism, calcium signaling, and hormone-mediated stress responses. Similarly, integration of genomic and phenomic data can identify genomic regions and molecular features associated with complex adaptive traits such as water-use efficiency, stay-green phenotype, and nutrient-use efficiency (Wang et al., 2023; Liu et al., 2026). Importantly, network inference based on correlation, co-expression, or statistical association does not by itself establish causality or the direction of regulatory relationships. A strong correlation between two molecular features may arise from direct regulation, indirect effects, shared upstream regulators, or common environmental responses. Therefore, inferred network edges should generally be interpreted as hypotheses of biological association unless supported by additional evidence. Causal modeling addresses a distinct objective by attempting to infer directional cause–effect relationships among variables. Approaches such as Bayesian networks, structural equation models, dynamic or time-resolved models, and other causal-inference frameworks can incorporate prior biological knowledge, temporal information, genetic variation, or experimental perturbation to evaluate candidate causal relationships. However, causal interpretation remains dependent on model assumptions and the quality of the available data and should ideally be supported by independent experimental validation. Thus, correlation-based network inference is useful for discovering candidate modules and regulatory relationships, whereas causal modeling provides a framework for testing or prioritizing directional regulatory hypotheses (Huang et al., 2025; Yan et al., 2026).

### 13.3 Technical challenges in multi-omics data integration

Although great strides have been made, there are technical limitations that constrain the effective integration of multi-omics. Omics datasets may be created across several experimental platforms, with different analytical pipelines and sampling protocols, which can lead to high batch effects (unwanted technical variation). Data comparability and reproducibility between independent studies is often hindered by variations in the sequencing technology, mass spectrometry platform, environment, sample type, developmental stage, and laboratory protocols. Therefore, there must be strong normalization procedures, batch correction algorithms, and cross-platform harmonization strategies that enable reliable biological interpretation. There is also a lack of standardized experimental and analytical workflows, which further hinders large-scale comparative analyses.

### 13.4 Computational infrastructure for predictive breeding

The adoption of the FAIR (Findable, Accessible, Interoperable and Reusable) principles for data is gaining increasing relevance for maximizing the value of multi-omics research. The management of data in a FAIR manner promotes open data sharing, metadata standardization, data identifiers and interoperability of databases, allowing for the re-use and integration of data produced by experiments conducted across various species of millets and experimental platforms (Wilkinson et al., 2016; Kundu and Tanti, 2026). At present, millet multi-omics datasets are distributed across individual studies and general-purpose or crop-oriented public databases rather than being consolidated in a single centralized, millet-specific multi-omics repository. Therefore, the establishment of a centralized millet-specific multi-omics repository should be regarded as a future infrastructure priority. Such a resource could facilitate standardized data deposition, cross-study integration, comparative analyses, collaborative breeding efforts, and reduce duplication of research efforts. Another challenge is the ever-growing need for more sophisticated computational infrastructure to process and integrate large-scale multi-omics data (Li et al., 2023; Sun et al., 2023). To efficiently process the complexity of systems biology analyses, high-performance computing, cloud-based storage platforms, scalable bioinformatics pipelines, and AI/ML algorithms will become increasingly important.

## 14 AI-assisted predictive breeding in millet crops

### 14.1 AI for multi-omics data integration and predictive breeding

AI is rapidly changing plant breeding from a descriptive to a predictive and data-driven science. AI offers unprecedented opportunities to combine complex multi-omics data with phenotyping and environmental information to rapidly develop superior cultivars in millet crops, a growing cereal crop recognized as a climate-resilient food source for marginal environments. While traditional breeding methods involve screening and selecting plants based on their phenotype or molecular markers, AI-driven predictive breeding utilizes genomics, transcriptomics, proteomics, metabolomics, phenomics, and environmental information to model complex genotype-to-phenotype relationships and enhance selection precision for complex traits like drought tolerance, heat stress, nutrient-use efficiency, grain quality, and yield stability. Among various AI methods, DL algorithms are among the most potent tools that can be used to uncover the underlying patterns in massive biological data. Artificial neural networks, CNNs, Recurrent Neural Networks (RNNs), and hybrid DL architectures can integrate genomic markers, transcript abundance, metabolite profiles, UAV-derived imagery, hyperspectral data, and environmental variables to model complex genotype-to-phenotype relationships. However, their apparent predictive advantage over conventional statistical or ML models depends strongly on sample size, feature dimensionality, experimental design, and validation strategy and therefore requires rigorous independent testing before broad application in millet breeding (van Dijk et al., 2021).

### 14.2 Deep learning, graph neural networks, and transformer architectures

Another exciting area of predictive breeding is GNNs, as biological systems are naturally interdependent and connected as a network. GNNs can be used to model gene regulatory networks, protein-protein interactions, metabolic pathways and molecular interaction networks simultaneously to identify important regulatory hubs that regulate stress adaptation processes. For millets, a combination of genomic variation and transcriptomic, proteomic, and metabolomic interaction networks that can be learned using graph-based approaches could be useful to identify candidate genes, functional pathways, and regulatory modules linked to abiotic stress tolerance. These network-aware methods may improve biological interpretability and, in some datasets, predictive performance by incorporating relationships among genes, proteins, metabolites, and genomic variants (Sun et al., 2023). However, their additional complexity may increase the risk of overfitting when millet datasets are small, and their performance should therefore be compared with simpler baseline models using independent and cross-environment validation. The architectures based on transformers designed for natural language processing are becoming relevant in the field of biological sequence analysis and multi-modal integration. Their self-attention mechanisms allow efficient modeling of long-range interactions in the genome and complex dependencies between different layers of the omics data.

### 14.3 LLM and foundation models for knowledge-driven crop improvement

Large Language Models (LLMs) have been found to be useful knowledge integration tools for crop improvement. While LLMs do not replace the need for genome-wide models for genomic prediction, they can significantly aid in breeding processes by summarizing scientific literature, annotating candidate genes, explaining multi-omics datasets, generating biological hypotheses, helping with experimental design and aiding in decision-making during breeding programs. The combination of data from published literature, genomic databases, phenotypic repositories, and breeding information can enable LLM-powered platforms to save time in knowledge discovery and help prioritize the deployment of candidate genes linked to millet crops’ climate resilience. Foundation models are the next generation of AI systems that can learn to represent biological information universally from extremely large and diverse sets of data.

### 14.4 AI-based decision support systems and challenges

AI is also a pillar of those AI-based breeding decision support systems which combine molecular data with agronomic knowledge. Current decision support systems integrate genomic prediction, high-throughput phenotyping, weather and crop simulation models with environmental monitoring to help breeders select parents, plan crosses, allocate resources, manage field trials and improve breeding populations (van Dijk et al., 2021). The addition of IoT sensors, UAVs, remote sensing platforms and cloud analytics further support real-time monitoring of crop performance and allow for dynamic optimization of the breeding programs at various locations. Despite these advances, several obstacles continue to limit the widespread adoption of AI-assisted predictive breeding for millet improvement. Although most species of millets have relatively small and scattered multi-omics datasets, this still hinders the training of complex AI models (Gao et al., 2025). However, applying AI to millet multi-omics data requires careful consideration of dataset size, model complexity, and validation. Many millet studies involve relatively small numbers of genotypes, environments, biological replicates, or multi-omics samples, while molecular datasets contain a large number of features. This high-dimensional, low-sample-size setting increases the risk of overfitting, particularly with DL, GNNs, and transformer-based models (Tsega and Mullualem, 2026). Therefore, high accuracy from random data partitioning may not reflect robust predictive performance. Feature selection, dimensionality reduction, regularization, early stopping, and simpler benchmark models can help control model complexity, while preprocessing, feature selection, and hyperparameter optimization should be restricted to the training data to prevent information leakage. Independent validation is also essential to assess model reliability and generalizability (Mishra et al., 2023). Validation should include independent genotypes or breeding populations, different years, locations, and stress conditions where possible. Cross-environment prediction is particularly challenging because environmental variation and genotype × environment interactions can alter molecular profiles and phenotypic expression. Thus, multi-environment trials, leave-one-environment-out validation, and incorporation of environmental covariates can provide more realistic estimates of model transferability (Zagre et al., 2025). For millet breeding, standardized multi-omics datasets linked to replicated phenotypes across environments will be important for the reliable deployment of complex AI models. AI predictions should therefore complement, rather than replace, independent experimental and field validation.

## 15 Translational breeding pipelines in millet crops

### 15.1 Gene discovery and genomic resource development

Although much progress has been made in the field of genomics and other omics technologies, the translation of molecular discoveries to better millet cultivars is still a huge challenge. Most of the studies have been directed towards finding the genes, QTLs or biomarkers that are responsive to stress under controlled conditions, while relatively few have been able to successfully incorporate those findings into practical breeding pipelines (Sun et al., 2023). To realize the future of millet improvement, an integrated translational breeding system needs to be developed that connects the basic molecular level research with cultivar development by using multi-omics, predictive breeding, genome editing and field testing. The first step in the translational pipeline is the discovery of the genes that are associated with drought, heat, salinity, nutrient-use efficiency, and other adaptive traits, using high throughput genomic approaches such as whole-genome sequencing, pan-genomics, GWAS, QTL mapping and comparative genomics (Shrestha et al., 2023).

### 15.2 Multi-omics validation and candidate gene prioritization

Genomic approaches such as GWAS, QTL mapping, and comparative genomics first identify genomic regions and candidate genes associated with stress-adaptive traits. These candidate genes can then be prioritized by integrating transcriptomics, proteomics, metabolomics, ionomics, epigenomics, and phenomics to determine whether the associated genomic variants are supported by changes in gene expression, protein abundance, metabolic profiles, elemental homeostasis, and phenotypic responses across relevant environmental conditions (Yang et al., 2021). Such multi-omics integration helps distinguish biologically relevant candidates from genes located within associated genomic regions and provides stronger evidence for their functional relevance. The prioritized candidates can subsequently be subjected to experimental or functional validation, including gene expression analysis, transgenic approaches, or genome editing, to establish their contribution to stress tolerance (Xue et al., 2026).

### 15.3 AI-assisted genomic prediction and precision genome editing

Genomic selection can potentially accelerate breeding progress by enabling the early selection of individuals based on predicted breeding values, thereby reducing the need for extensive phenotypic evaluation before advancement. However, the extent to which it reduces the duration of breeding cycles depends on factors such as prediction accuracy, the quality and relevance of the training population, trait architecture, generation turnover, and the integration of genomic prediction with rapid generation advancement and field validation. Thus, genomic selection should be viewed as a tool that can improve selection efficiency and potentially shorten breeding timelines rather than as a universal means of greatly reducing breeding-cycle duration (Crossa et al., 2017; Jarquin et al., 2020; Sajja et al., 2025).

### 15.4 Multi-environment field validation and cultivar deployment

Molecular validation alone is insufficient for cultivar development. Multi-environment field validation is an essential process for assessing the stability, adaptability and agronomic performance of improved breeding lines in realistic production environments. With the availability of high-throughput field phenotyping platforms such as UAVs, hyperspectral imaging, thermal sensors, LiDAR and IoT monitoring, it is possible to accurately evaluate the interaction between genotype, environment, and management. These phenotypic data, when combined with genomic prediction models, greatly enhance selection efficiency and enable molecular gains to be realized under field conditions (Araus and Cairns, 2014; Tattaris et al., 2016; Crossa et al., 2017). The last phase is cultivar release and deployment, which includes national performance tests, seed multiplication, quality evaluation, varietal registration, and release to farmers of genetically improved millet cultivars. Molecular breeding should be coupled with participatory varietal selection, socio-economic evaluation, climate adaptation studies and development of seed systems at this stage to ensure optimal adoption and impact. Efficient integration of genomic discoveries with predictive analytics, precision phenotyping, genome editing and rigorous crop field testing will therefore be critical for the success of future millet improvement programs.

## 16 Future research directions

Millets have proven to be crops that are resilient to climate change and have immense potential to help meet food and nutrition security in an uncertain climate. Although remarkable progress has been made in the fields of genomics, transcriptomics, proteomics, metabolomics, phenomics and epigenomics, the vast majority of research has concentrated on individual omics layers or specific stress conditions, making it difficult to gain a comprehensive understanding of the highly complex networks of molecular components involved in stress adaptation. Future millet studies should therefore be focused on systems biology rather than descriptive molecular characterization, which will lead to predictive and integrative systems biology studies with a direct connection to precision breeding and cultivar development (Varshney et al., 2020; Yang et al., 2021). Future millet improvement can be envisioned as a gradual transition over several phases of millet improvement that are interconnected. The first phase laid the groundwork for developing basic genomic resources such as reference genomes, QTLs, GWAS and molecular markers for the major millet species. These resources significantly fueled the progress of gene discovery, but most of them gave one-size-fits-all information about genetic variation. In the second phase, molecular knowledge grew by providing molecular function data, like transcriptomics, proteomics, metabolomics, epigenomics, ionomics and high-throughput phenomics (Sun et al., 2023).

Combining genomic, transcriptomic, proteomic, metabolomic, epigenomic, microbiome and phenomic data simultaneously will help to reconstruct gene regulatory networks and systems-level models of stress adaptation. Single-cell omics, spatial transcriptomics, long-read sequencing, pan-genomics, graph genomes and temporal multi-omics studies should be given special attention in future research to reflect the tissue specific and developmental changes in response to multiple abiotic stresses. Such integrative approaches will facilitate identification of strong biomarkers, causal genes, and hubs that control complex adaptive responses (Liu et al., 2021; Varshney et al., 2021). AI and ML will be the fourth step in this roadmap that will translate big data with multi-omics into biological knowledge. Accelerating candidate gene prioritization, biomarker discovery, phenotype prediction, and genomic selection with DL, GNNs, transformer-based architectures, explainable AI, and genomic prediction models. There are opportunities to use AI with high-throughput phenotyping, remote sensing, UAVs, and IoT-based environmental monitoring to assess interactions of genotype × environment × management in real-time, thus reducing breeding efficiency (Crossa et al., 2022). In addition to predictive analytics, digital twin technology is a groundbreaking opportunity for millet improvement. Digital Twins are a dynamic virtualization of a crop genotype that incorporates multi-omics, physiological, phenotypic, environmental and management data to simulate crop growth and reactions to stress across a range of agroecological scenarios.

## 17 Conclusion

Millets are climate-resilient crops with exceptional potential to enhance global food and nutritional security under changing environmental conditions. However, the complex genetic architecture of abiotic stress tolerance limits the effectiveness of conventional breeding and single-omics approaches. This review highlights that integrating genomics, transcriptomics, proteomics, metabolomics, epigenomics, ionomics, and high-throughput phenomics provides a systems-level understanding of stress adaptation by linking genetic variation with molecular regulation, physiological responses, and agronomic performance. Recent advances in pangenomics, long-read sequencing, single-cell and spatial omics, AI, and high-throughput phenotyping are transforming stress biology from descriptive analyses to predictive breeding. The integration of multi-omics with genomic selection, GWAS, and genome editing offers unprecedented opportunities to identify superior alleles, accelerate candidate gene validation, and develop climate-resilient millet cultivars. Nevertheless, major challenges remain, including limited integration of heterogeneous datasets, insufficient multi-environment field validation, lack of standardized analytical pipelines, and the gap between molecular discoveries and practical breeding applications. Future research should prioritize integrated multi-stress investigations, AI-assisted data integration, systems biology, and predictive genotype-to-phenotype models to improve breeding efficiency under diverse agroecological conditions.
